# Non-asymptotic Properties of Individualized Treatment Rules from Sequentially Rule-Adaptive Trials

**Published:** 2022

**Authors:** Daiqi Gao, Yufeng Liu, Donglin Zeng

**Affiliations:** Department of Statistics and Operations Research, The University of North Carolina at Chapel Hill Chapel Hill, NC 27599, USA; Department of Statistics and Operations Research, Department of Genetics, Department of Biostatistics, The University of North Carolina at Chapel Hill Chapel Hill, NC 27599, USA; Department of Biostatistics, The University of North Carolina at Chapel Hill Chapel Hill, NC 27599, USA

**Keywords:** Contextual bandit, empirical process, martingale, outcome weighted learning, sequential decision making

## Abstract

Learning optimal individualized treatment rules (ITRs) has become increasingly important in the modern era of precision medicine. Many statistical and machine learning methods for learning optimal ITRs have been developed in the literature. However, most existing methods are based on data collected from traditional randomized controlled trials and thus cannot take advantage of the accumulative evidence when patients enter the trials sequentially. It is also ethically important that future patients should have a high probability to be treated optimally based on the updated knowledge so far. In this work, we propose a new design called sequentially rule-adaptive trials to learn optimal ITRs based on the contextual bandit framework, in contrast to the response-adaptive design in traditional adaptive trials. In our design, each entering patient will be allocated with a high probability to the current best treatment for this patient, which is estimated using the past data based on some machine learning algorithm (for example, outcome weighted learning in our implementation). We explore the tradeoff between training and test values of the estimated ITR in single-stage problems by proving theoretically that for a higher probability of following the estimated ITR, the training value converges to the optimal value at a faster rate, while the test value converges at a slower rate. This problem is different from traditional decision problems in the sense that the training data are generated sequentially and are dependent. We also develop a tool that combines martingale with empirical process to tackle the problem that cannot be solved by previous techniques for i.i.d. data. We show by numerical examples that without much loss of the test value, our proposed algorithm can improve the training value significantly as compared to existing methods. Finally, we use a real data study to illustrate the performance of the proposed method.

## 1. Introduction

For many diseases, patients respond heterogeneously to treatments and a one-size-for-all strategy is often not effective. Recent technology advances allow personalized treatment suggestions by tailoring it to patient characteristics, including demographics, medical histories or genetic information (Hamburg and Collins, 2010). The personalized policy is often referred to as the Individualized Treatment Rule (ITR), which aims to maximize a predefined reward such as the patient’s health status.

The optimal ITR can be estimated through regression-based or classification-based methods. The former fits a regression model for the rewards and finds the treatment with the maximum estimated reward (Qian and Murphy, 2011). The latter obtains the optimal ITR directly by maximizing the average reward. For example, Zhao et al. (2012) proposed a weighted classification algorithm called outcome weighted learning (OWL), which is based on the support vector machine (SVM) and equipped with various kernels. There are also variations of OWL designed for ITR estimation in single-stage problems (Zhou et al., 2017; Chen et al., 2018) and multi-stage problems (Zhao et al., 2015; Liu et al., 2018).

For all the above methods, to avoid unobserved confounding bias as present in observational studies, data used to learn optimal ITRs are typically obtained from randomized controlled trials (RCTs), where patients receive treatments based on a prefixed probability rule. RCTs are conducted primarily to compare the efficacy of new treatments. However, in the case when the control drug is not beneficial or is even harmful, patients may have to switch treatments or withdraw from the study due to little benefit or adverse events under the assigned treatments. This may cause violation of the randomization and result in bias in estimating clinical efficacy. In fact, as data are gathered during the process, we already have an inference about which treatment should be better for the next patient. A more effective design for the trial should be sequentially adaptive so that any new patients entering the trial are more likely to receive the best treatment learned from the past. This is especially important ethically since an inferior treatment may cause severe health issues to a patient. A sequentially adaptive trial has the advantage to better maintain randomization while keeping most of the study participants benefiting from their assigned treatments. As commented in Thall (2002), a clinical trial ideally should provide patients in the trial with the best treatment available, while also generate data for improving therapies. We will discuss the tradeoff between the two goals from a statistical viewpoint in this paper. We refer to the clinical trial data as the training set and refer to an independent population as the test set for clarity.

The clinical trials that allow the trial protocol to be modified according to observed patient information as the trial continues are called adaptive clinical trials (ACTs) (Chow, 2014). A special class of ACTs is the response-adaptive randomization (Hu and Rosenberger, 2006), which is divided into four categories: restricted randomization, response-adaptive randomization, covariate-adaptive randomization, and covariate-adjusted response-adaptive (CARA) randomization. The latter three are adjusted for response, covariates, and response with covariates respectively. As an example of CARA, Zhang et al. (2007) proposed a framework for the treatment distribution to converge to a predefined distribution, which can be applied to generalized linear models. Hu et al. (2015) suggested to balance ethics in avoiding assigning patients to inferior arms and efficiency in the power of detecting treatment differences. ACTs sometimes also use Bayesian designs to find the optimal dose schedule based on efficacy and toxicity and maximize survival time by combining different phases (Thall et al., 2013; Riviere et al., 2018; Chapple and Thall, 2019). These methods mainly use adaptive designs to improve the efficiency, which refer to the power of estimating average treatment effects, and are not suitable for learning optimal ITRs. There are also a few papers for learning subgroup treatment effects through enrichments (Kim et al., 2011; Lai et al., 2012; Renfro et al., 2016), but they are not optimal for finding ITRs. Furthermore, theoretical justification is lacking for the estimated treatment effects for all subgroups.

There is a close connection between the sequentially adaptive design and the contextual bandit, which is a class of algorithm that deals with online decision problems. As a single-stage special case of reinforcement learning, it aims at making sequential decisions through trial and error. All reinforcement learning algorithms encapsulate an “exploration-exploitation” dilemma. Various exploration methods have been proposed in the contextual bandit literature. The ϵ-greedy methods assign the current optimal arm with a probability of 1-ϵ or chooses from all arms randomly with a total probability of ϵ (Yang and Zhu, 2002; Chen et al., 2020). Boltzmann exploration assigns probabilities of whether to follow the current optimal policy using the soft-max function based on the estimated mean rewards of arms (Sutton and Barto, 2018). Upper-Confidence Bound (UCB) methods choose the arm with the largest upper confidence bound, which either has a large estimated mean reward or a large estimated variance (implying great uncertainty) (Li et al., 2010; Chu et al., 2011; Krause and Ong, 2011). Bayesian methods assign a treatment to a future patient according to the posterior distribution of reward parameters (Chapelle and Li, 2011; Liao et al., 2020). Action elimination is another branch that ignores the inferior arms gradually (Perchet and Rigollet, 2013). Different estimation methods have also been proposed in linear scenarios (Auer, 2002; Li et al., 2010; Chu et al., 2011; Chen et al., 2020; Bastani and Bayati, 2020) and nonlinear scenarios (Yang and Zhu, 2002; Krause and Ong, 2011; Zhou et al., 2020) under the contextual bandits framework. Interested readers are referred to Tewari and Murphy (2017); Lattimore and Szepesvári (2020) for a comprehensive review of bandit problems. However, most works in contextual bandits focus on the training phase and do not address the test performance theoretically. Pure exploration with a fixed budget in multi-armed bandits (MAB, Lattimore and Szepesvári (2020)) also tries to minimize the test regret (also called simple regret), but they generally do not require a small training regret (also called cumulative regret). Bubeck et al. (2009) illustrated the tradeoff between training and test performance in MAB algorithms without a context, that an asymptotically optimal policy for training regret will lead to a suboptimal policy for test regret. Lattimore and Szepesvári (2020) also discussed in Chapter 33 that algorithms with logarithmic cumulative regret in MAB settings (for example UCB) are not well suited for pure exploration. In contrast, we consider more complex settings with context and also provide a way to find the balance point.

To our best knowledge, the most relevant clinical trial design for learning ITR is the active clinical trial (Minsker et al., 2016), which is an active-learning based algorithm. In terms of data collection, they focus on exploring patients close to the decision boundary and omit the trials on patients known to benefit from one of the treatments with a high probability. However, the actually conducted trials are still purely randomized, and will not benefit from previous information. Practically speaking, the patients omitted from the trial still need to be recruited to collect their basic information before deciding whether they are close to the boundary, which can still create a burden on the trial and the patients.

We propose a sequentially adaptive trial design named “rule-adaptive design” in contrast to “response-adaptive design”. It updates the treatment assignment policy during the clinical trial using some statistical or machine learning methods, so that the outcomes in the clinical trial are improved. In the meantime, we also allow for some exploration probability in order to learn an efficient final ITR. In the current work, we consider estimating the two-armed ITR with OWL and explore with ϵ-greedy or a variation of Boltzmann exploration. Different from most contextual bandit methods which rely on a regression model of the rewards, our OWL-based algorithm is a weighted classification method which tries to maximize the rewards directly. Only a model for the treatment effect is specified and thus minimum assumption (for example, boundedness) is needed for the main effect, unlike in Li et al. (2010) and Chen et al. (2020) where a reward model is constructed for the total effect. While Chambaz et al. (2017) and Chen et al. (2020) focused on the inference of the parameters or value functions, we perform the regret analysis.

Specifically, we consider a trial with n sequentially enrolled patients with independent feature variables. Since some of the characteristics of a patient can only be observed after the patient is enrolled in the clinical trial and the process maybe expensive, we assume that we cannot choose which patients to enroll. After a pilot trial of some patients, we assign any incoming patient the estimated optimal treatment learnt from the available data with a probability of p and the other treatment with a probability of 1-p. We restrict that p is bounded by 1-ϵ and ϵ, where ϵ is a positive constant between 0 and 0.5. Furthermore, we let ϵ decay to zero as the ITR estimation gets more accurate over the trial. If the probability p is a constant that does not depend on the current context or the history information, including the characteristics, treatments and rewards of previous patients, the above method is actually ϵ-greedy. Note that the ϵ defined here is one half of that in the definition of ϵ-greedy in most reinforcement learning literature. However, we allow p to be dependent on the current status and the history in theory and in simulation. In this algorithm, p governs the chance of exploration. Intuitively, a small p indicates a high tendency to follow the current estimated ITR. Future patients to enter the trial are likely to receive a favorable treatment when data accumulate. On the other hand, a small p limits the chance of exploring new treatments. This leads to a slow convergence of the learnt ITR to the optimal one, yielding a suboptimal ITR if the training sample size in the trial is not large enough. This suggests a tradeoff between training and test performance. Our proposed class of algorithms allows adaptive probabilities to depend on already collected data in a flexible way, and includes Boltzmann exploration and an approximate UCB algorithm as special cases.

In this paper, to fully characterize the performance of the rule-adaptive design, we establish the convergence rate of both the test regret for the learnt ITR if implemented in an independent population, and the training regret for patients in the training set. The former concerns the expected reward loss as compared to the theoretically optimal ITR. The latter describes the cumulative reward loss between actually observed rewards and the hypothetical rewards if each patient would receive the learnt optimal ITR over time. The established bounds depend on the number of initial patients, the number of patients enrolled in the main trial, and the decay rate of the ϵ-sequence. The bounds clearly indicate a tradeoff between the training and test performance of the algorithm. This tradeoff can be useful for us to choose an ϵ-sequence that guarantees a small loss of rewards for the testing sample due to the reduction of exploration in the training process, while at the same time allowing a majority of the experiment patients to receive better than random treatments. To our knowledge, these are the first rigorous results for contextual bandits.

Our proofs for establishing bounds are substantially different from the ones that are based on i.i.d. training data, due to the challenge that the treatment assignment depends on the past data. In the proof, we derive a new concentration inequality for suprema of a martingale sequence by extending the results in Rakhlin et al. (2015). Particularly, to obtain the sequential Rademacher complexity of function classes needed in the inequality, we develop a new mathematical tool that applies the empirical process and bracketing number technique to martingale sequences. Bae and Levental (1995) showed that Freedman’s inequality (Freedman, 1975) works well for ergodic Markov chains as a substitution for Bernstein’s inequality in i.i.d. sequences. Van de Geer (1995), Nishiyama (1997) and Nishiyama et al. (2000) also took similar approaches in continuous-time martingales or some martingales with jumps. Rakhlin et al. (2015) created a scheme of extending empirical process and symmetrization methods to martingale. Chambaz et al. (2017) derived a new maximal inequality for martingales based on the uniform entropy integral. However, to our knowledge, our paper is the first one to make use of bracketing numbers in the test value bound of martingale sequences. As a remark, we note that Rakhlin and Sridharan (2014) provided a bound for sequential Rademacher complexity of linear functions on dual spaces of covariates and linear coefficients. In contrast, our method applies to any function class with bounded bracketing integral.

The rest of this paper is organized as follows. In Section 2, we describe our proposed algorithm that uses the OWL algorithm for learning ITRs over time. Section 3 gives theoretical guarantees for the performance of our algorithm on the training and test sets. We describe the implementation details of our proposed algorithm, and discuss the connections and differences between our algorithm and existing methods in Section 4. In Section 5, we conduct extensive simulation studies to examine how parameters in our algorithm influence the empirical results, and compare our method with randomized controlled trials, LinUCB (Li et al., 2010) and active clinical trials (Minsker et al., 2016). We further use a real data example to illustrate the advantage of the proposed method in Section 6. The paper is concluded with some remarks in Section 7.

## 2. Methodology

We consider the single-stage decision problem, the case where a single treatment recommendation is made for every patient. For each patient, the feature variables or covariates $X\in 𝒳\subset R^{d}$ are observed. We assume that the covariates ${\left\{X_{i}\right\}}_{i=1}^{\infty }$ are drawn from a population independently and identically. Based on the covariates, we need to decide which treatment to take for the patient. We focus on a two-armed problem in this paper. That is, the treatment A takes values in 𝒜={1,-1}. An outcome R∈R is then observed, which is also called the reward, with higher values desirable. An ITR is a map 𝒟:𝒳↦𝒜 that assigns the patient of covariates X to a treatment A. An optimal ITR can generate the largest mean reward for the test data. If there exists a measurable discriminant function f:𝒳↦R such that 𝒟=sign⁡{f}, we only need to find such a function f.

### 2.1 Learning Algorithm for Updating ITRs

We propose to estimate the ITR using machine learning methods, OWL in particular, since it is shown to provide useful ITR recommendations in various scenarios (Zhao et al., 2012). We briefly describe the method of OWL below.

Let P be the joint distribution of Z:=(X,A,R) and E be the corresponding expectation. If the data are sampled according to the ITR 𝒟, that is, given A=𝒟(X), the distribution and expectation are denoted as $P^{𝒟}$ and $E^{𝒟}$ respectively. Then the optimal ITR can be defined as ${𝒟}^{*}:=\text{arg}{\text{max}}_{𝒟}\;E^{𝒟}(R)$ and the optimal decision function $f^{*}$ satisfies $\text{sign}\left\{f^{*}\right\}={𝒟}^{*}$. Qian and Murphy (2011) showed that the expected reward under policy 𝒟 is given by

(1) $$E^{𝒟}(R)=E\left[\frac{R1(A=𝒟(X))}{\pi (A;X)}\right],$$

where π(A;X) is the probability of taking treatment A given covariates X of a patient. After transforming (1) to a loss function based on the 0–1 loss, Zhao et al. (2012) proposed OWL to instead minimize a surrogate loss, hinge loss $\phi (x)=[1-x{]}^{+}$. That is, they try to find the function f that minimizes $E\left[g^{f}(Z)\right]$, where $g^{f}(Z)=R\phi (Af(X))/\pi (A;X)$. If we obtain a total number of n observations, OWL tries to minimize

$$\frac{1}{n}\overset{n}{\underset{i=1}{\sum }}\frac{R_{i}}{\pi_{i}\left(A_{i};X_{i}\right)}\phi \left(A_{i}f\left(X_{i}\right)\right).$$

A penalty term can be added to the loss function for high-dimensional settings to avoid overfitting. This is a weighted classification problem that can make use of the framework of SVM. The estimated ITR can be obtained by taking $\overset{ˆ}{D}=\text{sign}\{\overset{ˆ}{f}\}$. The resulting estimator of ITR generated by OWL is consistent (Zhao et al., 2012). Moreover, $R_{i}$ can be replaced by $R_{i}-E\left(R|X_{i}\right)$ to further improve the learning performance (Liu et al., 2018).

### 2.2 Sequentially Rule-Adaptive Trials (SRATs)

We describe the proposed algorithm to improve the clinical trial outcome and learn the optimal ITR as follows. Before the trial begins, assume we already have a pure randomized pilot trial of small size $n_{0}$, from which our first function ${\overset{ˆ}{f}}_{0}$ can be estimated. Then the first patient i=1 can choose to follow ${\hat{𝒟}}_{0}$ or not. The observations in initial samples all have a propensity score of 0.5. The function is updated after each patient has been treated. Denote the estimated function based on data before the ith patient coming as ${\overset{ˆ}{f}}_{i-1}$, and the corresponding ITR as ${\hat{𝒟}}_{i-1}$ for i=1,…,n. Assume $p_{i}$ is a probability that can depend on the current feature variables $X_{i}$ and the history information of previous patients, bounded away from 0 and 1 for all i. At each time point i, we choose to follow our current estimated ITR ${\hat{𝒟}}_{i-1}$ with a probability $p_{i}$ or choose the other treatment with a probability $1-p_{i}$. Let $I_{i}$ be a binary variable such that the ith treatment follows ${\hat{𝒟}}_{i-1}$ if $I_{i}=1$ and follows $-{\hat{𝒟}}_{i-1}$ if $I_{i}=-1$. That is, $I_{i}$ takes the value 1 with a probability of $p_{i}$ and the value −1 with a probability of $1-p_{i}$. Then the treatment can be chosen as $A_{i}=I_{i}{\hat{𝒟}}_{i-1}\left(X_{i}\right)$.

When $p_{i}$ only depends on the order i but not on the history and covariates, our algorithm actually follows the ϵ-greedy exploration method. Note that the randomization probability is sometimes described in another way. In most reinforcement learning literature, for ${\tilde{\epsilon }}_{i}\in (0,1]$ at stage i,ϵ-greedy chooses the best arm with a probability of $1-{\tilde{\epsilon }}_{i}$; and with a total probability of ${\tilde{\epsilon }}_{i}$, it chooses from all arms randomly with equal probability. Our definition coincides with this in the sense that $1-p_{i}={\tilde{\epsilon }}_{i}/2$. We use the slightly different notation here to describe the boundedness assumption of $p_{i}$ in a more general way. In the special case when $p_{i}=0.5$ for all i=1,…,n, the adaptive clinical trial degenerates into a purely randomized clinical trial. Besides, as a limiting case without truncation, Boltzmann exploration assumes $p_{i}={\text{logit}}^{-1}(\text{benefit}{\;}_{i})$, where $\text{benefit}\;{}_{i}$ is the difference between the estimated rewards of two treatments for the patient i.

Although we choose the presumed best arm with a high probability, there is also some chance that we explore the other arm and observe consequences. When i is small, estimations are usually not accurate due to the large sampling bias and estimation bias. A large probability should be assigned to the inferior arm to allow for exploration and reduce variances. When data accumulate and the ITR estimation gets more accurate as i increases, we will take a higher probability for following the current estimated ITR. Therefore, a decreasing sequence of ${\left\{p_{i}\right\}}_{i=1}^{n}$ is desirable. As n goes to infinity, we want $p_{n}\to 0$ if our estimation method is consistent. The speed at which $p_{n}$ decreases depends on the convergence rate of estimation.

Let $Z_{i}^{\left(0\right)}=\left\{X_{i}^{\left(0\right)},A_{i}^{\left(0\right)},R_{i}^{\left(0\right)},I_{i}^{\left(0\right)}\right\}$ be the feature variables, treatments and rewards of the patient $i=1,\ldots ,n_{0}$ in the pilot trial. Here $I_{j}^{\left(0\right)}$ can take any value since we do not have an estimated ITR to follow in the pilot trial. Similarly, denote $Z_{i}=\left\{X_{i},A_{i},R_{i},I_{i}\right\}$ to be all the information about the patient i=1,…,n in the main trial. Extend the definition of $P,P^{𝒟}$ and $E,E^{𝒟}$ to be the joint distributions and expectations of Z respectively. For simplicity, denote $H_{i-1}$ as the history information for the ith patient, where $H_{0}:=\left\{Z_{1}^{\left(0\right)},Z_{2}^{\left(0\right)},\ldots Z_{n_{0}}^{\left(0\right)}\right\}$ and $H_{i}:=\left\{Z_{1}^{\left(0\right)},Z_{2}^{\left(0\right)},\ldots Z_{n_{0}}^{\left(0\right)},Z_{1},Z_{2},\ldots ,Z_{i}\right\},i=1,\ldots ,n$. Then before we decide which treatment to take for the ith (i=1,…,n) patient, the data that we can base our decision on are $\left\{H_{i-1},X_{i}\right\}$. The final ITR is estimated from the whole training sample $H_{n}$.

To adapt the algorithm of estimating ITRs to our sequential setting, we will denote $\pi_{i}\left(A_{i};H_{i-1},X_{i}\right)$ as the probability of taking treatment $A_{i}$ at stage i to indicate that it depends on the history $H_{i-1}$ and the covariates $X_{i}$ for the main trial. The probability $p_{i}=p_{i}\left(H_{i-1},X_{i}\right)$ defined as $P\left(I_{i}=1|H_{i-1},X_{i}\right)$ also depends on the history and covariates, and is a simplified notation for $\pi_{i}\left({\hat{𝒟}}_{i-1}\left(X_{i}\right);H_{i-1},X_{i}\right)$.

We make the following assumptions to quantify potential outcomes for both the pilot trial and the main trial. Although data are sequentially generated in the main trial, $R_{i}$ still only depends on $A_{i}$ and $X_{i}$ for each i=1,…,n.

**Assumption 1 (Ignorability)** *The treatment* $A_{i}\left(A_{i}^{\left(0\right)}\right)$ *is independent of the potential outcome* $R_{i}^{*}(a)\left(R_{i}^{(0)*}(a)\right)$ *given feature variables* $X_{i}\left(X_{i}^{\left(0\right)}\right)$ *for all* a∈𝒜 *and all* $i=1,\ldots ,n\left(i=1,\ldots ,n_{0}\right)$.

**Assumption 2 (Consistency)** *The observed outcome* $R_{i}\left(R_{i}^{\left(0\right)}\right)$ *under a treatment* $A_{i}=a\left(A_{i}^{\left(0\right)}=a\right)$ *equals the potential outcome* $R_{i}^{*}(a)\left(R_{i}^{(0)*}(a)\right)$ *for all* a∈𝒜 *and all* $=1,\ldots ,n\;\left(i=1,\ldots ,n_{0}\right)$.

For the pilot trial, we make an additional assumption on propensity scores.

**Assumption 3 (Positivity)** *There exists a constant* $c_{0}>0$ *such that* $\pi_{i}\left(a;X_{i}^{\left(0\right)}\right)\geq c_{0}$ *for all* a∈𝒜 *and all* $X_{i}^{\left(0\right)}\in 𝒳$ *for all* $i=1,\ldots ,n_{0}$.

We do not need to make the positivity assumption for the main trial since it is guaranteed by our data generating process when we require $p_{i}\left(H_{i-1},X_{i}\right)$ to be bounded away from 0 and 1. We will formally quantify the assumptions on the probability $p_{i}\left(H_{i-1},X_{i}\right)$ in Sections 3.1 and 3.2. Different choices of $p_{i}\left(H_{i-1},X_{i}\right)$ will be discussed in Section 4 and their performances will be compared in Section 5

Following the scheme of OWL, we propose to minimize the ϕ-risk using the hinge loss in a function class ℱ:𝒳↦R. Using OWL, we can obtain the first estimated function

(2) $${\hat{f}}_{0}=\underset{f\in ℱ}{\text{argmin}}\frac{1}{n_{0}}\overset{n_{0}}{\underset{j=1}{\sum }}\frac{R_{j}^{\left(0\right)}}{\pi_{j}\left(A_{j}^{\left(0\right)}\right)}\phi \left(A_{j}^{\left(0\right)}f\left(X_{j}^{\left(0\right)}\right)\right)$$

using the pilot trial and update it to get

(3) $${\hat{f}}_{i}=\underset{f\in ℱ}{\text{argmin}}\frac{1}{n_{0}+i}\left\{\overset{n_{0}}{\underset{j=1}{\sum }}\frac{R_{j}^{\left(0\right)}}{\pi_{j}\left(A_{j}^{\left(0\right)}\right)}\phi \left(A_{j}^{\left(0\right)}f\left(X_{j}^{\left(0\right)}\right)\right)+\overset{i}{\underset{j=1}{\sum }}\frac{R_{j}}{\pi_{j}\left(A_{j};H_{j-1},X_{j}\right)}\phi \left(A_{j}f\left(X_{j}\right)\right)\right\}$$

for i=1,…,n along with the main trial. For weighted SVM problems, the function class ℱ is generally taken to be a linear space for linear decision rules or a reproducing kernel Hilbert space (RKHS) for nonlinear decision rules. The full algorithm is summarized in Algorithm 1.

## 3. Theoretical Results for SRAT

In order to demonstrate a tradeoff between training and test performance of our algorithm, we need to bound the estimated value function on both sets. Previous work has shown a bound for the test value trained on i.i.d. data (Zhao et al., 2012). We will expand the bounds of OWL to dependent training samples.

### 3.1 Performance Guarantee for the Test Set

Define $𝒱(f):=E^{\text{sign}\{f\}}(R)$ as the value function of f. We use value function $𝒱\left({\overset{ˆ}{f}}_{n}\right)$ as an indicator of how well our algorithm performs on the test set, after training on n observations. We will call $𝒱\left({\overset{ˆ}{f}}_{n}\right)$ the test value, and define $𝒱\left(f^{*}\right)-𝒱\left({\overset{ˆ}{f}}_{n}\right)$ as the test regret. Remember that $𝒱\left(f^{*}\right)={\text{max}}_{f}\;𝒱(f)$ according to the definition of $f^{*}$. In this paper we assume that the optimal function $f^{*}$ belongs to the function class ℱ, in which we find the estimated ITR. As a consequence, Zhao et al. (2012, Theorem 3.2) implies that the excess risk satisfies $0\leq E^{\text{sign}\left\{f^{*}\right\}}(R)-E^{\text{sign}\{f\}}(R)\leq E\left[g^{f}(Z)\right]-E\left[g^{f^{*}}(Z)\right]$. If f minimizes $E\left[g^{f}(Z)\right]$, the righthand side cannot be larger than zero since $f^{*}$ is also in the function class ℱ. Therefore, we have $E^{\text{sign}\{f\}}(R)=E^{\text{sign}\left\{f^{*}\right\}}(R)$, which suggests that f and $f^{*}$ have the same Bayesian risk. For example, we would reasonably assume that $f^{*}$ is linear in the covariates or in the basis function of covariates for a linear space ℱ. The following result shows that the test regret converges in probability and gives the convergence rate.


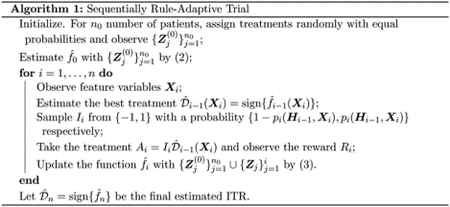


We first introduce some key notations. With $N_{\left[\right]}(\eta ,ℱ,∥\cdot ∥)$ being the bracketing number for the set ℱ with respect to the semi-norm ∥⋅∥, define a bracketing integral of ℱ as

$$J_{\left[\right]}\left(\delta ,ℱ,∥\cdot ∥\right):=\int_{0}^{\delta }\sqrt{1+\text{log}N_{\left[\right]}\left(\eta ,ℱ,∥\cdot ∥\right)}\text{d}\eta .$$

Let $L_{2}(P)$ norm be the $L_{2}$ norm with respect to measure P. An envelope of function class ℱ is any function F:𝒳↦R such that f(x)≤F(x) for every x∈𝒳 and f∈ℱ. The minimal envelope function is $F(x)={\text{sup}}_{f\in ℱ}\;|f(x)|$, for all x∈𝒳. The * symbols on the top right corner of P and E indicate outer probability and the corresponding outer expectation respectively in order to avoid measurability problems (Van der Vaart and Wellner, 1996).

**Assumption 4** *Suppose we have a nonincreasing sequence of* $\left\{\epsilon_{1},\ldots ,\epsilon_{n}\right\}$ *with* $\epsilon_{i}\in (0,0.5]$ *for all* i=1,…,n, *where each* $\epsilon_{i}$ *can only depend on the order* i. *Assume* $\epsilon_{i}\leq p_{i}\left(H_{i-1},X_{i}\right)\leq 1-\epsilon_{i}$ *almost surely for all* i.

**Assumption 5** *There exists a positive constant* r *such that* ${∥R_{i}∥}_{\infty }\leq r$ *for all* i.

**Assumption 6** *Suppose* ℱ *is a class of measurable functions satisfying*

(4) $$\int_{0}^{\infty }\sqrt{1+\log\;N_{\left[\right]}\left(\eta ,ℱ,L_{2}\left(P\right)\right)}d\eta \;<\;\infty .$$

*Let* F *be the minimal envelope function of* ℱ *and assume* F *has a weak second moment, that is,* $x^{2}P^{*}(F(X)>x)\to 0$ *as* x→∞.

**Theorem 1** *Assume the pilot trial satisfies Assumptions 1, 2, 3, 5 and the main trial satisfies Assumptions 1, 2, 4, 5. If we take* $c_{0}=0.5$ *and a function class satisfying Assumption 6 in* Algorithm 1, *then with a probability higher than* $1-e^{-\delta }$ *for any* δ>0,

(5) $$𝒱\left(f^{\text{*}}\right)-𝒱\left({\hat{f}}_{n}\right)\leq \frac{C}{n_{0}+n}\left[\left(J+\sqrt{\delta b}\right)r\sqrt{n_{0}}+rb\delta +\frac{r^{2}bJ}{\epsilon_{n}^{2}}\sqrt{\delta n{\text{log}}^{3}n}\right],$$

where $J:={\text{sup}}_{P}\;J_{\left[\right]}\left(∥F{∥}_{P,2},ℱ,L_{2}(P)\right),b:={\text{sup}}_{f\in ℱ}\;∥f{∥}_{\infty }$, and C is a constant depending on δ,r,b,J and ${\left\{\epsilon_{i}\right\}}_{i=1}^{n}$.

**Remark 2** *The above bound shows that the terms containing* $n_{0}$ *are not dominant as long as the order of* $n_{0}$ *does not exceeds the order of* n, *since* $\epsilon_{n}$ *is nonincreasing and* $\epsilon_{n}^{-2}$ *has an order of* Ω(1). *In practice,* $n_{0}$ *can be taken as the minimum value that a stable initial rule* ${\overset{ˆ}{f}}_{0}$ *can be estimated with. For example, if the covariates* X *has a dimension d including an intercept,* $n_{0}$ *can be taken as* d+1 *for linear kernel. We choose* $n_{0}$ *to be a small constant in our simulation study in* Section 5. *For generality, we will assume that* $n_{0}=O(n)$ *in the following analysis, which includes the constant* $n_{0}$ *as a special case.*

**Remark 3** *Note that the bracketing number and covering number here are defined for i.i.d. data, since* ℱ *is defined on* 𝒳 *and the observed feature variables* ${\left\{X_{i}\right\}}_{i=1}^{\infty }$ *are i.i.d. The constant* J *characterizes the complexity of the function class* ℱ. *It generally increases as the dimension* d *of covariates increases, and will result in a larger upper bound.*

**Remark 4** *For the bound (5) to be non-trivial, we need the right-hand side to be* $o_{p}(1)$. *That is, when assuming* $n_{0}=O(n)$, *we need* $\epsilon_{n}$ *to decay slower than* $n^{-1/4}{\text{log}}^{3/4}(n)$ *and* J *to be finite. Intuitively speaking, if the* ϵ *sequence decays too fast and the algorithm is extremely greedy in the training process, then the data sample is biased and cannot be used to learn an efficient final ITR.*

**Remark 5** *Theorem 1 holds when* n *is large enough but finite, so Assumption 4 ensures that the positivity assumption is satisfied for all* n. *The randomness parameter* $\epsilon_{n}$, *which can be close to zero, is incorporated in the error bounds and accounts for the variance inflation in the value estimation. Simulation study in* Section 5 *shows that there is no significant variance inflation for different choices of* $\epsilon_{n}$ *sequences in practice. The complexity of the function class containing* $\pi_{i}$ *increases as the lower and upper bounds of propensity score get wider, but our proof only relies on the lower bound* $\epsilon_{i}$.

In our sequentially dependent algorithm, any constant sequence $\left\{\epsilon_{1},\ldots ,\epsilon_{n}\right\}$ can generate a convergence rate of $n^{-1/2}{\text{log}}^{3/2}(n)$ as long as $n_{0}=O(n)$. If we take $\epsilon_{i}=0.5$ for all i, the algorithm degenerates to pure randomization. Therefore, the traditional RCT is actually a special case contained in our framework. Zhao et al. (2012) proved that the convergence rate of OWL with the Gaussian kernel almost achieves $n^{-1/2}$ under the Geometric noise assumption. The extra ${\text{log}}^{3/2}(n)$ term comes from a martingale concentration inequality that we used, as shown in Section C in the supplementary material. This indicates that the efficiency of learning ITR is not significantly affected by using sequentially generated data.

**Example 1** *If* ℱ *is a class of linear functions with bounded parameters* $\beta \in \text{ℬ}\subset R^{d}$, *the above assumptions are satisfied. Linear functions are Lipschitz in parameters in the sense that* $\left|f_{\beta_{1}}(x)-f_{\beta_{2}}(x)\right|\leq m\left(\beta_{1},\beta_{2}\right)G(x)$ *for Euclidean metric* m *on the index parameter set*, $G(x)=∥x{∥}_{2}$, *and for every* $\beta_{1},\beta_{2}$ *by Cauchy-Schwarz inequality. By Theorem 2.7.11 of* Van der Vaart and Wellner (1996), $N_{\left[\right]}(2\eta ∥G∥,\text{ℱ},∥\cdot ∥)$ *is bounded by* N(η,ℬ,m). *Since* $N(\eta ,\text{ℬ},m)\leq K/\eta^{d}$ *for some constant* $K,N_{\left[\right]}\left(\eta ,\text{ℱ},L_{2}(P)\right)$ *can be bounded by* $2^{d}K∥G{∥}_{P,2}^{d}/\eta^{d}$ *for all measure* P. *If we further assume that* $∥G{∥}_{P,2}\leq u$ *for all measure* P *and some constant* u>0, *for example, when the covariate space* 𝒳 *is bounded, then the constant* J *and the integral in (4) is finite. The assumptions in Theorem 1 are then satisfied.*

The general idea of proof is to find a classification risk bound for the weighted SVM on sequentially generated data. It is quite similar to the proof idea of Theorem 4 in Bartlett et al. (2006). However, the key step of their proof relies on a variant of Talagrand’s inequality (Talagrand, 1994; Bousquet, 2002), which is a concentration inequality of suprema of empirical process on i.i.d data. On the contrary, our algorithm generates data that are adapted to a filtration.

We will define some new notations here. For any sequence ${\left\{Y_{i}\right\}}_{i\in N}$ adapted to a filtration ${\left\{{𝒢}_{i}\right\}}_{i\in N}$, observe that ${\left\{E_{i-1}f\left(Y_{i}\right)-f\left(Y_{i}\right)\right\}}_{i\in N}$ is a martingale difference sequence for any measurable function f, where $E_{i-1}(\cdot ):=E\left(\cdot |{𝒢}_{i-1}\right)$. Define a martingale process indexed by f∈ℱ analogous to an empirical process as

$$f\mapsto M_{n}\left(f\right):=\frac{1}{n}\overset{n}{\underset{i=1}{\sum }}\left\{E_{i-1}f\left(Y_{i}\right)-f\left(Y_{i}\right)\right\}.$$

In accordance with Rakhlin et al. (2015), the scaling factor $\sqrt{n}$ is not included in the definition.

In our setting, let ${𝒢}_{0}=\sigma \left\{H_{0}\right\}$ and ${𝒢}_{i}=\sigma \left\{H_{i}\right\},i\in N$, so that ${\left\{Z_{i}\right\}}_{i\in N}$ is adapted to the filtration ${\left\{{𝒢}_{i}\right\}}_{i\in N}$. Similar as the definition in Section 2.1, let the loss function on a single observation in a sequential experiment be

$$g^{f}\left(Z_{i}\right)=\frac{R_{i}\phi \left(A_{i}f\left(X_{i}\right)\right)}{\pi_{i}\left(A_{i};H_{i-1},X_{i}\right)}.$$

Note that $Z_{i}$ is implicitly dependent on the history $H_{i-1}$ through $A_{i}$. Define $h^{f}\left(Z_{i}\right)=g^{f}\left(Z_{i}\right)-g^{f^{*}}\left(Z_{i}\right)$ as the difference between the loss generated by any f and the optimal function $f^{*}$. Based on our weighted classification setting, we can further define a weighted version of the martingale process by

$$W_{n}\left(f\right):=M_{n}\left(h^{f}\right)=\frac{1}{n}\overset{n}{\underset{i=1}{\sum }}\left[E_{i-1}h^{f}\left(Z_{i}\right)-h^{f}\left(Z_{i}\right)\right].$$

The key step is to bound the test regret by the conditional expectations of $h^{f}$. To extend the idea to a martingale sequence, we make use of sequential complexity techniques and a suprema concentration inequality presented in Rakhlin et al. (2015, Lemma 13). The inequality essentially relies on $E{\text{sup}}_{f\in ℱ}\;WV_{n}(f)$, so we first present the following lemma for the upper bound of the expectation of suprema. We use the symbol “≲” to indicate that the left-hand side is no larger than the right-hand side for all n up to a universal constant.

**Lemma 6** *Assume the main trial satisfies Assumptions 1, 2, 4, 5. If we take a function class satisfying Assumption 6 in* Algorithm 1, *then*

(6) $$E\underset{f\in ℱ}{\text{sup}}W_{n}\left(f\right)≲\frac{r}{\sqrt{n}\epsilon_{n}}J_{\left[\right]}\left(∥F{∥}_{\mathbb{P},2},ℱ,L_{2}\left(\mathbb{P}\right)\right).$$

The above lemma suggests that if some f performs well enough on the training set compared to $f^{*}$, then it should not be too bad on the test set as well. When $\epsilon_{n}$ does not depend on $n,E{\text{sup}}_{f\in ℱ}\;W_{n}(f)$ converges at a rate of $n^{-1/2}$, which is the same as the rate for independent data.

### 3.2 Performance Guarantee for the Training Set

We propose to use ${\overset{‾}{R}}_{n}:=\sum_{i=1}^{n}\;R_{i}/n$ as the measure of performance on the training set, which does not concern the pilot trial. We will call ${\overset{‾}{R}}_{n}$ the training value and it indicates what we really observe in n patients drawn out of the population. Furthermore, we define our regret on the training set as $\sum_{i=1}^{n}\;\left[𝒱\left({\overset{ˆ}{f}}_{i-1}\right)-R_{i}\right]/n$. Each observed reward $R_{i}$ is compared with the corresponding $𝒱\left({\overset{ˆ}{f}}_{i-1}\right)$, which is the value function based on previous (i-1) data points, and the sum of differences is recorded.

A common metric in bandit problems for training data is the cumulative regret for n observations. It is defined as the difference between the expectation of the sum of rewards under the optimal ITR and that under the estimated ITR, that is, $\sum_{i=1}^{n}\;E_{x_{i}}R\left({𝒟}^{*}\left(x_{i}\right)\right)-E_{x_{i}}R\left({\hat{𝒟}}_{i-1}\left(x_{i}\right)\right)$, where $x_{i}$ is the instantiated tailoring variable vector for the ith (i=1,…,n) patient. It mainly measures how much benefit the actual treatments generate compared with the optimal ones for fixed tailoring variables $\left(x_{1},\ldots ,x_{n}\right)$ regardless of the randomness in rewards. A bound on the expectation of regret or a probably approximately correct (PAC) bound is often derived. However, the true optimal rule ${𝒟}^{*}$ and the expectation of rewards are unknown in the training process. Furthermore, the cumulative regret does not include the intrinsic randomness in rewards.

Here we present the training regret bound in terms of our definition with an additional assumption on the randomization probability $p_{i}$.

**Assumption 7** *Suppose we have another nonincreasing sequence of* $\left\{\epsilon_{1}^{\prime },\ldots ,\epsilon_{n}^{\prime }\right\}$ *with* $\epsilon_{i}^{\prime }\in (0,1)$ *for all* i=1,…,n, *where each* $\epsilon_{i}^{\prime }$ *can only depend on the order* i. *Assume that* $p_{i}\left(H_{i-1},X_{i}\right)\geq 1-\epsilon_{i}^{\prime }$ *almost surely for all* i.

Under Assumptions 4 and 7, the two sequences ${\left\{\epsilon_{i}\right\}}_{i=1}^{n}$ and ${\left\{\epsilon_{i}^{\prime }\right\}}_{i=1}^{n}$ actually help create upper and lower bounds of $1-p_{i}\left(H_{i-1},X_{i}\right)$, which are needed in the test and training regret bounds respectively.

**Theorem 7** *Assume the main trial satisfies Assumptions 1, 2, 5, 7 and we have* ${0<p}_{i}\left(H_{i-1},X_{i}\right)<1$ *for all* i. *Then with a probability higher than* $1-e^{-\delta }$ *for any* δ>0,

(7) $$\left|\frac{1}{n}\overset{n}{\underset{i=1}{\sum }}\left[𝒱\left({\hat{f}}_{i-1}\right)-R_{i}\right]\right|\leq C^{\prime }r\left[\sqrt{\frac{\delta \wedge \delta^{2}}{n}}+\left(\frac{1}{n}\overset{n}{\underset{i=1}{\sum }}\epsilon_{i}^{\text{'}}\right)\right],$$

where $C^{\prime }$ is a constant depending on δ,r and ${\left\{\epsilon_{i}^{\prime }\right\}}_{i=1}^{n}$.

**Remark 8** *The concentration bound implies that the training regret is upper bounded by the average of the* $\epsilon_{i}^{\prime }$ *sequence plus a term of order* $O_{p}(1/\sqrt{n})$. $\epsilon_{i}^{\prime }$ *should be of order* o(1) *if we need the training regret to converge to zero. Otherwise, if there is always some probability that the inferior treatment is taken, the training reward cannot be optimal. Specifically, when* ${\left\{\epsilon_{i}^{\prime }\right\}}_{i=1}^{n}$ *is constant and does not rely on* i, *the above bound is a constant. The purely randomized clinical trial is a special case of this setting.*

**Remark 9** *In most of the cases, the randomization probability of following the inferior treatment is* $1-p_{i}\left(H_{i-1},X_{i}\right)\leq \epsilon_{i}^{\prime }\leq 0.5$ *and is nonincreasing. For example, Assumption 7 is satisfied by* ϵ-greedy with nonincreasing $\epsilon_{i}^{\prime }=\epsilon_{i}$ *for all* i. *However, in some special cases such as Boltzmann exploration,* $p_{i}\left(H_{i-1},X_{i}\right)$ *can be less than 0.5 if the estimated benefit is negative. This can happen when the method for learning ITR (OWL in our design) and that for estimating benefit (for example, linear regression) are different. While OWL recommends* ${\hat{𝒟}}_{i-1}\left(X_{i}\right)$, *the difference between the estimated rewards of* ${\hat{𝒟}}_{i-1}\left(X_{i}\right)$ and $-{\hat{𝒟}}_{i-1}\left(X_{i}\right)$ *can be negative. In this case, we also need the probability of a negative benefit to converge to zero at a certain rate.*

If we assume the true optimal value function $𝒱\left(f^{*}\right)$ is known and compare each $R_{i}$ for i=1,…,n with it, we have the following result. Note that $\left|𝒱\left(f^{*}\right)-{\overset{‾}{R}}_{n}\right|$ is a notion more similar to the cumulative regret. Except for the randomness in rewards, the difference only lies in the optimal value. While the cumulative regret considers maximum rewards for each individual, we still focus on the population value.

**Corollary 10** *Let the assumptions in Theorems 1 and 7 hold. With a probability higher than* $1-e^{-\delta }$ *for any* δ>0,

$$\left|𝒱\left(f^{\text{*}}\right)-{\bar{R}}_{n}\right|\leq C^{\prime \prime }[r\sqrt{\frac{\delta \wedge \delta^{2}}{n}}+r\left(\frac{1}{n}\overset{n}{\underset{i=1}{\sum }}\epsilon_{i}^{\text{'}}\right)+\frac{1}{n\left(n_{0}+i\right)}\overset{n-1}{\underset{i=0}{\sum }}(\left(J+\sqrt{\left(\delta +\text{log}n\right)b}\right)r\sqrt{n_{0}}+rb\left(\delta +\text{log}n\right)+\frac{r^{2}bJ}{\epsilon_{i}^{2}}\sqrt{i{\text{log}}^{3}i\left(\delta +\text{log}n\right)})],$$

where $C^{\prime \prime }$ is a constant depending on r,b,J,δ and the sequences ${\left\{\epsilon_{i}\right\}}_{i=1}^{n},{\left\{\epsilon_{i}^{\prime }\right\}}_{i=1}^{n}$, if we take $i{\text{log}}^{3}i=0$ for i=0.

The above corollary demonstrates the well-known exploration-exploitation tradeoff in contextual bandits when the observed reward is compared to the true optimal value. The first two terms on the left-hand side come from Theorem 7, which characterize the loss in the value due to exploration and increases as $\epsilon_{i}^{\prime }$ increases. On the other hand, the last term, which comes from Theorem 1, describes the regret of exploiting the estimated ITR compared to the optimal ITR and decreases with more exploration. The optimal rate is achieved when the two components strike a balance.

### 3.3 Tradeoff Between Training and Test Values

In this section, we discuss the tradeoff between the training value and the test value. To better describe the convergence rates of training and test values, we can set a decreasing schedule for $\epsilon_{n}$ and $\epsilon_{n}^{\prime }$. Here we assume $\epsilon_{n}$ and $\epsilon_{n}^{\prime }$ decreases polynomially with n since the upper bounds in (5) and (7) are dominated by polynomial terms of n.

**Theorem 11** *Assume* $\epsilon_{n}=\epsilon_{0}n^{-(1-\theta )/4}$ *with* $\epsilon_{0}\in (0,0.5],\theta \in (0,1]$ *and* $\epsilon_{n}^{\prime }=\epsilon_{0}^{\prime }n^{-\left(1-\theta^{\prime }\right)/4}$ *with* $\epsilon_{0}^{\prime }\in (0,1),\theta^{\prime }\in (-\infty ,1]$. *Let Assumptions 1.7 hold and assume* $\epsilon_{n}\leq \epsilon_{n}^{\prime }$ *for all* n. *If* $n_{0}=O(n)$, *then the test value* $𝒱\left({\overset{ˆ}{f}}_{n}\right)$ *converges to* $𝒱\left(f^{*}\right)$ *at a rate of* $O_{p}\left(n^{-\theta /2}(\text{log}n{)}^{3/2}\right)$, *and the training value* $\sum_{i=1}^{n}\;R_{i}/n$ *converges to* $\sum_{i=1}^{n}\;𝒱\left({\overset{ˆ}{f}}_{i-1}\right)/n$ *at a rate of* $O_{p}\left(n^{-\left(1-\theta^{\prime }\right)/4}\right)$. *If we further assume that* $\theta =\theta^{\prime }$ *and* $\epsilon_{0}\leq \epsilon_{0}^{\prime }$, *then the two regrets converge at the same rate* $O_{p}\left(n^{-1/6}\right)$ *when* θ=1/3.

The above results suggest that the convergence rate in the logarithmic scale is negative in θ for the test regret and positive in $\theta^{\prime }$ for the training regret. When θ and $\theta^{\prime }$ are close to $0,{\left\{\epsilon_{i}\right\}}_{i=1}^{n}$ and ${\left\{\epsilon_{i}^{\prime }\right\}}_{i=1}^{n}$ decay fast and the algorithm is greediest on the training set, leading to a fast convergence of the training value and a slow convergence of the test value. On the contrary, when $\theta =\theta^{\prime }=1,{\left\{\epsilon_{i}\right\}}_{i=1}^{n}$ and ${\left\{\epsilon_{i}^{\prime }\right\}}_{i=1}^{n}$ are constant sequences that does not change with the order i. The test value converges quickly while the training value may not converge in this case. This demonstrates in theory why there is a tradeoff between training and test values. Where the “balance” point is can be defined differently in difference settings. Theorem 11 provides a balance point where the two rates match with each other. In Section 5 and 6, we will further demonstrate the tradeoff between training and test values using numerical examples. Note that ϵ-greedy satisfies the assumptions with $\theta =\theta^{\prime }$ and $\epsilon_{0}=\epsilon_{0}^{\prime }$.

## 4. Implementation

Recall that in theory we allow the randomization probability $p_{i}\left(H_{i-1},X_{i}\right)$ to be a constant or be dependent on the current covariates $X_{i}$ and the history $H_{i-1}$. In implementation, when $p_{i}\left(H_{i-1},X_{i}\right)$ is a constant that only depends on the order i, the exploration method becomes the special case ϵ-greedy. We call the full algorithm SRAT-E in this case.

To build a bridge between ϵ-greedy and UCB methods, for example LinUCB (Li et al., 2010) in linear cases, we propose to let $p_{i}\left(H_{i-1},X_{i}\right)$ depend on the history in the following way. While OWL provide an estimation of ITR, we need a separate regression model to show how much benefit a patient will gain from one treatment against the other. In the case of a greatly positive benefit, we can assign the current patient ${\hat{𝒟}}_{i-1}\left(X_{i}\right)$ with a large probability since we are almost sure that this treatment is the better one. On the contrary, if the benefit is negative, we allow for more exploration. Specifically, let ${\overset{ˆ}{\mu }}_{a}\left(H_{i-1},X_{i}\right)$ and ${\overset{ˆ}{\sigma }}_{a}\left(H_{i-1},X_{i}\right)$ be the estimated mean and standard deviation of the reward of the ith patient given the treatment a, where $a\in \left\{{\hat{𝒟}}_{i-1}\left(X_{i}\right),-{\hat{𝒟}}_{i-1}\left(X_{i}\right)\right\}$. Denote ${\overset{ˆ}{U}}_{a}\left(\alpha_{i},H_{i-1},X_{i}\right)={\overset{ˆ}{\mu }}_{a}\left(H_{i-1},X_{i}\right)+\alpha_{i}{\overset{ˆ}{\sigma }}_{a}\left(H_{i-1},X_{i}\right)$ as the upper confidence bound of the estimated reward, where $\alpha_{i}$ is a constant tuning parameter that does not depend on ${𝒢}_{i-1}$ or $X_{i}$. Note that the estimations rely on the regression model completely, since OWL does not provide an estimation of rewards, but only provides a distance between the covariate point and the decision boundary. Further define

$${\hat{B}}_{i}\left(\alpha_{i},H_{i-1},X_{i}\right)={\hat{U}}_{{\hat{𝒟}}_{i-1}\left(X_{i}\right)}\left(\alpha_{i},H_{i-1},X_{i}\right)-{\hat{U}}_{-{\hat{𝒟}}_{i-1}\left(X_{i}\right)}\left(\alpha_{i},H_{i-1},X_{i}\right)$$

as the UCB-based benefit, which is the difference between the estimated UCB of rewards given two treatments. Let the probability $p_{i}$ be

$$p_{i}\left(H_{i-1},X_{i}\right)=\{\begin{array}{ll} 1-\epsilon_{i} & \text{ if }{\hat{B}}_{i}\left(\alpha_{i},H_{i-1},X_{i}\right)\geq 0,\\ \text{max}\left\{\epsilon_{i},{\text{logit}}^{-1}\left\{\frac{{\hat{B}}_{i}\left(\alpha_{i},H_{i-1},X_{i}\right)}{\gamma_{i}}\right\}\right\} & \text{ if }{\hat{B}}_{i}\left(\alpha_{i},H_{i-1},X_{i}\right)<0 \end{array},$$

where $\gamma_{i}$ is a constant tuning parameter that does not depend on ${𝒢}_{i-1}$ or $X_{i}$. Recall that we truncate the probability by $\epsilon_{i}$ because we require that $p_{i}$ is bounded away from 0 and 1. We call this method SRAT-B since the randomization probability is partially based on Boltzmann exploration.

In practice, we can estimate ${\overset{ˆ}{\mu }}_{a}$ and ${\overset{ˆ}{\sigma }}_{a}$ for a∈𝒜 by ${\overset{ˆ}{\mu }}_{a}\left(H_{i-1},X_{i}\right)=X_{i}^{T}{\overset{ˆ}{\beta }}_{a}\left(H_{i-1}\right)$ and ${\overset{ˆ}{\sigma }}_{a}\left(H_{i-1},X_{i}\right)={\left[X_{i}^{T}{\overset{ˆ}{W}}_{a}{\left(H_{i-1}\right)}^{-1}X_{i}\right]}^{1/2}$, where ${\overset{ˆ}{\beta }}_{a}\left(H_{i-1}\right)$ and ${\overset{ˆ}{W}}_{a}\left(H_{i-1}\right)$ are the estimated linear parameter and variance matrix before stage i. Following Li et al. (2010, Algorithm 1), the initial estimates can be obtained by

$${\hat{W}}_{a}\left(H_{0}\right)=I_{d}+{\left(X_{a}^{\left(0\right)}\right)}^{T}X_{a}^{\left(0\right)},\;{\hat{Y}}_{a}\left(H_{0}\right)={\left(X_{a}^{\left(0\right)}\right)}^{T}R_{a}^{\left(0\right)},\;\text{and}\;{\hat{\beta }}_{a}\left(H_{0}\right)={\hat{W}}_{a}{\left(H_{0}\right)}^{-1}{\hat{Y}}_{a}\left(H_{0}\right),$$

where

$$X_{a}^{\left(0\right)}:={\left[X_{j}^{\left(0\right)}\right]}_{j:A_{j}^{\left(0\right)}=a}^{T}\text{ and }R_{a}^{\left(0\right)}:={\left[R_{j}^{\left(0\right)}\right]}_{j:A_{j}^{\left(0\right)}=a}^{T}$$

for all a∈𝒜. The identity matrix $I_{d}$ of dimension d is added to avoid the singularity of ${\overset{ˆ}{W}}_{a}$ when the sample size is small. Then we iteratively update ${\overset{ˆ}{\beta }}_{a}$ and ${\overset{ˆ}{W}}_{a}$ for $a=A_{i}$ after each stage i by

$${\hat{W}}_{a}\left(H_{i}\right)={\hat{W}}_{a}\left(H_{i-1}\right)+X_{i}X_{i}^{T},\;{\hat{Y}}_{a}\left(H_{i}\right)={\hat{Y}}_{a}\left(H_{i-1}\right)+R_{i}X_{i}$$

and let ${\overset{ˆ}{\beta }}_{a}\left(H_{i}\right)={\overset{ˆ}{W}}_{a}{\left(H_{i}\right)}^{-1}{\overset{ˆ}{Y}}_{a}\left(H_{i}\right)$. The parameters for the treatment not selected at the stage i, which is $a=-A_{i}$, will not be updated at this stage.

When ${\overset{ˆ}{B}}_{i}\left(\alpha_{i},H_{i-1},X_{i}\right)\geq 0$, it means that the regression model prefers ${\hat{𝒟}}_{i-1}\left(X_{i}\right)$ than $-{\hat{𝒟}}_{i-1}\left(X_{i}\right)$. This is also the conclusion by the OWL model. Therefore, we are actually requiring the treatment to follow ${\hat{𝒟}}_{i-1}$ with high probability when the two models agree with each other. However, when the two models disagree, we assign treatment $-{\hat{𝒟}}_{i-1}$ with a soft probability based on the estimated benefit.

In LinUCB, the treatment is taken as $\text{sign}\left\{{\overset{ˆ}{U}}_{1}\left(\alpha_{i},H_{i-1},X_{i}\right)-{\overset{ˆ}{U}}_{-1}\left(\alpha_{i},H_{i-1},X_{i}\right)\right\}$ with a probability 1, where the regression model is the ordinary least squares (OLS) model. This implies that $P\left(I_{i}=1\right)=P\left(A_{i}={\hat{𝒟}}_{i-1}\left(X_{i}\right)\right)=1\left[{\overset{ˆ}{B}}_{i}\left(\alpha_{i},H_{i-1},X_{i}\right)\geq 0\right]$. The actual value of ${\hat{𝒟}}_{i-1}\left(X_{i}\right)$ does not really matter here since the probability is symmetric for the two treatments. If ${\hat{𝒟}}_{i-1}\left(X_{i}\right)=1$, then

$$\mathbb{P}\left(A_{i}=1|H_{i-1},X_{i}\right)=1\left[{\hat{U}}_{1}\left(\alpha_{i},H_{i-1},X_{i}\right)-{\hat{U}}_{-1}\left(\alpha_{i},H_{i-1},X_{i}\right)\geq 0\right];$$

otherwise, if ${\hat{𝒟}}_{i-1}\left(X_{i}\right)=-1$, then

$$\mathbb{P}\left(A_{i}=-1|H_{i-1},X_{i}\right)=1\left[{\hat{U}}_{-1}\left(\alpha_{i},H_{i-1},X_{i}\right)-{\hat{U}}_{1}\left(\alpha_{i},H_{i-1},X_{i}\right)\geq 0\right]\;=1-\mathbb{P}\left(A_{i}=1|H_{i-1},X_{i}\right)$$

and they are equivalent if $P\left({\overset{ˆ}{U}}_{1}\left(\alpha_{i},H_{i-1},X_{i}\right)={\overset{ˆ}{U}}_{-1}\left(\alpha_{i},H_{i-1},X_{i}\right)\right)=0$.

**Figure 1:**
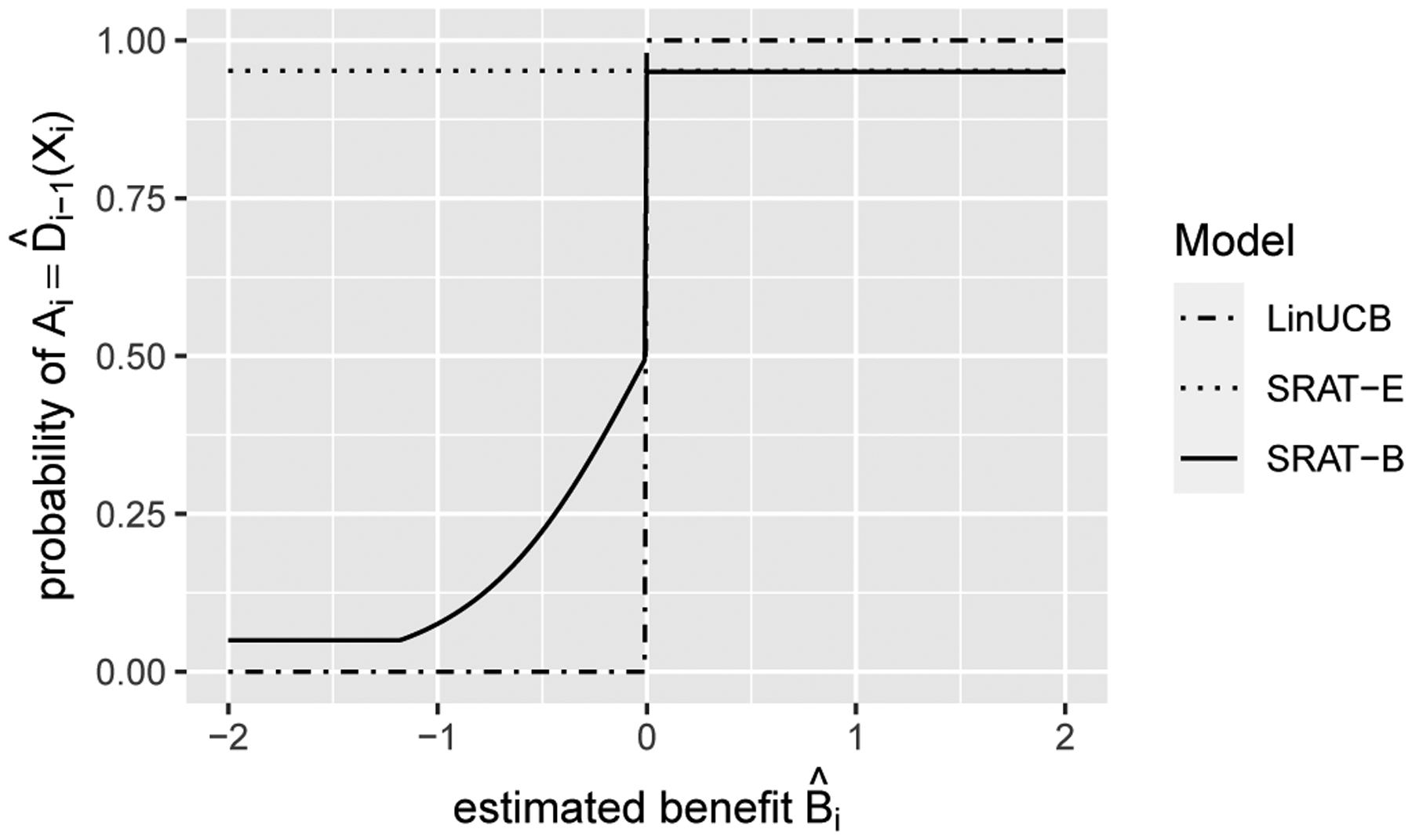
The randomization probability $P\left(A_{i}={\hat{𝒟}}_{i-1}\left(X_{i}\right)|H_{i-1},X_{i}\right)$ of SRAT-E, SRAT-B and LinUCB when $\epsilon_{i}=0.05$ and $\gamma_{i}=0.4$.

The relationship between SRAT-E, SRAT-B and LinUCB can be illustrated in Figure 1. While the randomization probability of SRAT-E is not affected by the estimated benefit of ${\hat{𝒟}}_{i-1}\left(X_{i}\right)$ over $-{\hat{𝒟}}_{i-1}\left(X_{i}\right)$, the probability of LinUCB is purely determined by this benefit. Note that the dot-dashed line is symmetric about zero for LinUCB, since the value of ${\hat{𝒟}}_{i-1}\left(X_{i}\right)$ does not affect the probability of $A_{i}=1$ as we discussed before. SRAT-B is a method that has an exploration probability in between, which actually approximates that of LinUCB when $\epsilon_{i}\to 0$ and γ→0. In this sense, our proposed variation of Boltzmann exploration is a soft version of UCB. If we also take OLS to be our model for estimating the benefit, we can view LinUCB as a limiting case of SRAT-B in the training process. However, since the treatment rules are learnt from OLS and OWL respectively, the test values are based on completely different final ITRs.

## 5. Simulation Study

We assess the empirical performance of SRAT on training and test samples using synthetic data. Here we examine two scenarios. In both scenarios, let X be a 10-dimensional vector $\left(X_{1},X_{2},\ldots ,X_{10}\right)$. Assume X has a joint distribution N(0,Σ) truncated by [-1,1]for each dimension, where Σ is the covariance matrix with 1 on the diagonal and 0.1 off-diagonal. The treatment A is generated from {-1,1} according to the SRAT algorithm and other algorithms to be compared. Assume the reward R is normally distributed with mean $Q_{0}(X,A)=m_{0}(X)+T_{0}(X,A)$ and variance $\nu_{0}(X)=0.2\left(X_{1}^{2}X_{3}+1\right)$. Here $m_{0}$ is the main effect and $T_{0}$ is the treatment effect. The variance $\nu_{0}$ is allowed to be a function of X to show that our proposed SRAT does not rely on the variance of rewards. We consider two scenarios as follows:

1. Linear treatment effect $T_{0}(X,A)=0.5\left(0.2-X_{1}-X_{2}\right)A$;
2. Nonlinear treatment effect $T_{0}(X,A)=0.5\left(0.2-X_{1}^{2}-X_{2}\right)A$.

In both scenarios, the main effect $m_{0}(X)=1+2X_{1}+X_{2}^{2}+2X_{2}X_{3}$ is nonlinear. It can be easily seen that the optimal ITR is determined by $T_{0}(X,A)$.

For our proposed SRAT algorithm, we first generate $n_{0}$ patients along with their purely randomized treatments and observed clinical outcomes. Then at each step i, we get a new sample of feature variables $X_{i}$, and estimate its current optimal treatment by ${\hat{𝒟}}_{i-1}$. In accordance with our theoretical results in Example 1, we only use the linear kernel for OWL. The package DTRlearn2 (Chen et al., 2019) is used to implement the OWL algorithm with $L_{2}$ penalty. It improves the learning performance by removing the main effect from the rewards and takes care of negative rewards by flipping the sign of the reward and the action simultaneously (Liu et al., 2018). Next, we sample the binary indicator $I_{i}$ with a probability $\left\{1-p_{i}\left(H_{i-1},X_{i}\right),p_{i}\left(H_{i-1},X_{i}\right)\right\}$ and take $A_{i}=I_{i}{\hat{𝒟}}_{i-1}\left(X_{i}\right)$. Here $p_{i}\left(H_{i-1},X_{i}\right)$ is defined in Section 4 for SRAT-E and SRAT-B differently, and the truncation parameter $\epsilon_{n}$ is defined as

$$\epsilon_{0}n^{-\left(1-\theta \right)/4},\text{ where }\epsilon_{0}\in \left(0,0.5\right],\theta \in \left(0,1\right]$$

as in Theorem 11. The final estimated ITR decision function ${\overset{ˆ}{f}}_{n}$ will be evaluated using the test data. We also include RCT, estimated by OWL, as a special case of SRAT with $\epsilon_{0}=0.5$ and θ=1 in our simulation.

To compare our algorithm with existing bandit methods in linear scenario, we also implement LinUCB (Li et al., 2010) for demonstration. It is widely used in reinforcement learning and its variation SupLinUCB (Chu et al., 2011) is known to be rate optimal in contextual bandit problems with linear reward functions. LinUCB chooses the treatment with the largest upper confidence bound of reward, which is estimated by linear regression. It does not require a pilot trial for initialization, but we still generate one of size $n_{0}$ for it in consistency with our algorithm.

The active clinical trial (Minsker et al., 2016) is also compared here, which targets an effective ITR. Minsker et al. (2016) applied the active learning technique in the clinical trial and proposed to only conduct clinical trials on patients close to the decision boundary. In this way, patients that will benefit from one of the treatments with a high probability can be omitted from the trial and thus save experiment expenses and efforts. Minsker et al. (2016) considered two nonparametric methods, Gaussian process regression (AL-GP) and kernel smoothing (AL-BV) to construct a confidence interval around the decision boundary. The actually recruited patients are assigned to each treatment with equal probabilities. Since the two methods generally perform similarly in different scenarios, we only compare with AL-GP in our simulation study. AL-GP also requires a pilot trial, and we take $n_{0}$ as the initial sample size as well.

We fit each estimation model of the corresponding algorithm with linear terms of $X_{1},\ldots ,X_{10}$ for scenario 1, and with both linear and quadratic terms of $X_{1},\ldots ,X_{10}$ for scenario 2. While SRAT-E, RCT, LinUCB and AL-GP involve only one model, SRAT-B relies on both OWL and OLS models. Since at least 21 observations are needed to fit an initial model with 20 predictors and an intercept for OWL in scenario 2, we choose $n_{0}=30$ for both scenarios, which is almost the least possible for a reliable estimate of the initial rule. By comparing different values of $n_{0}$, we see that $n_{0}$ does not affect the results of SRAT-E and SRAT-B significantly. Larger $n_{0}$ reduces randomness but does not improve the average performance. This verifies the theoretical result that $n_{0}$ is not a dominating term in the theoretical bound as long as it has an order O(n).

We have proved the properties of training and test regrets of SRAT in theoretical analysis and they will be used here as an indication of training and test performance of each algorithm. Each value function 𝒱 is computed numerically using a sample of size 100,000 randomly drew out of an independent population. The value function is estimated using the mean reward on this set.

**Figure 2:**
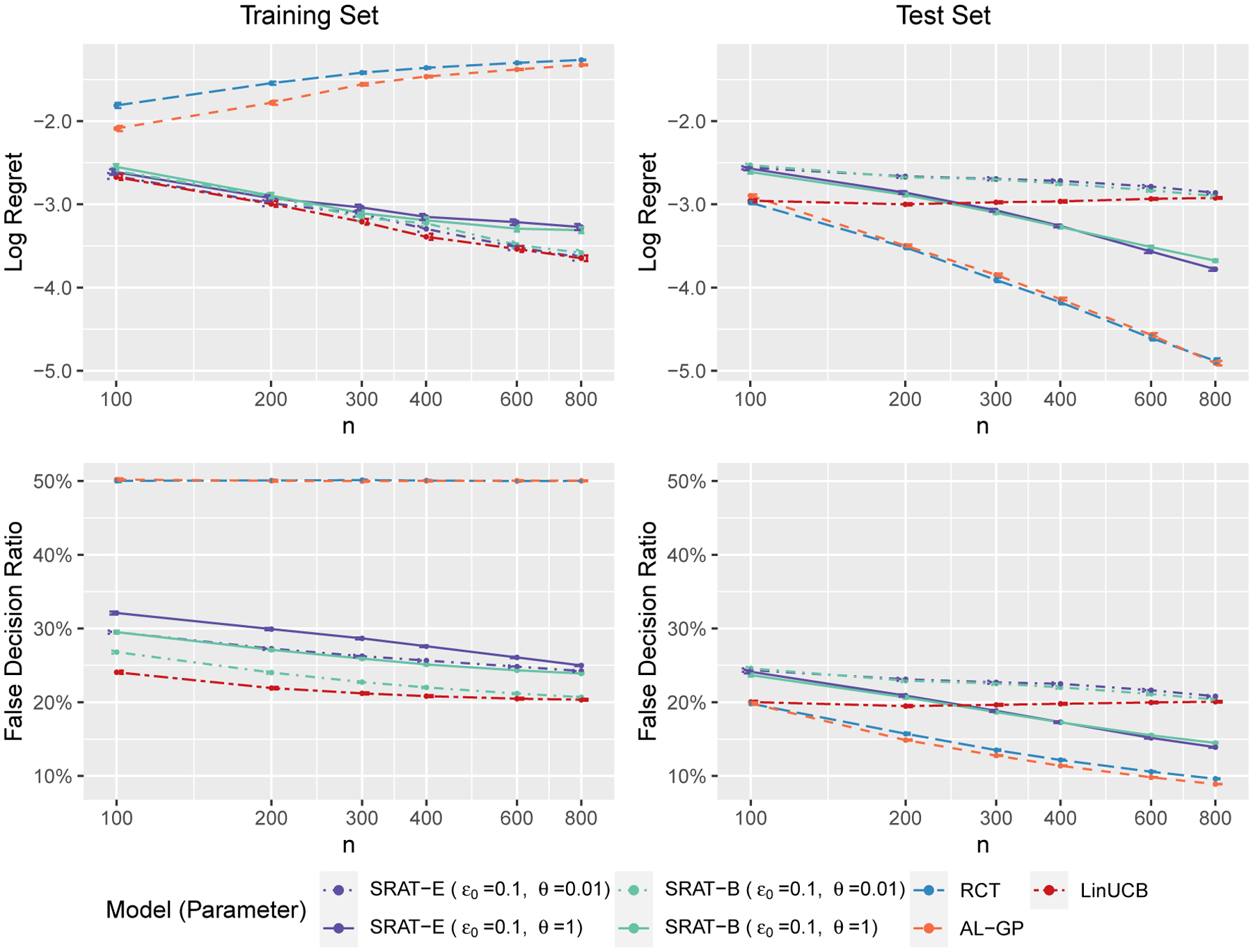
Scenario 1. The regret (logarithmic scale) and the false decision ratio on the training or test set against sample size n.

We first compare the convergence rate of regret for different algorithms. SRAT-E and SRAT-B are implemented with $\epsilon_{0}=0.1$ and θ=0.01 or 1. As will be discussed later in Figure 4, the training and test regrets are monotone in the parameters $\epsilon_{0}$ and θ. Therefore, to save space, we only show two possible combinations of parameters here. The scheduling parameter $\gamma_{i}$ for SRAT-B is taken as $0.999^{i}$ so that it will not decay too fast to zero. RCT is a special case of SRAT with $\epsilon_{0}=0.5$ and θ=1. According to Li et al. (2010), the clickthrough rate (mean reward) of LinUCB in news article recommendation does not change much on the deployment bucket (test set) when α≥0.2, while it decreases quickly on the learning bucket (training set) as α increases from 0.2. In our experiment settings, α does not affect training and test regrets significantly. Therefore, we will fix $\alpha_{i}=0.2$ for all i for LinUCB and SRAT-B in our following experiments. The process is repeated 1,000 times and the resulting values are averaged across all iterations. To better illustrate the polynomial relationship between training or test regret and the sample size n, we plot the regret values and the sample sizes on the logarithmic scale. The false decision ratio, or 1 – accuracy in classification literature, is also displayed against n. One standard error of the mean regret or the mean false decision ratio across the 1,000 iterations is reported on each point. The result of scenario 1 is plotted in Figure 2. The plot of scenario 2, Figure 7, is included in the supplementary material since it shows a similar conclusion as scenario 1.

According to Figure 2, LinUCB is the greediest on the training process, with the least regret and false decision ratio. As discussed in Section 4, LinUCB can actually be viewed as a limiting case of SRAT-B on the training set. Indeed, our proposed greediest algorithms, SRAT-E and SRAT-B with parameters $\epsilon_{0}=0.1$ and θ=0.01, perform similarly as LinUCB in terms of training regret. AL-GP and RCT take purely randomized treatments on the training set, so they have the largest training regret and a 50% training accuracy. Since the training regret is calculated based on $𝒱\left({\overset{ˆ}{f}}_{n-1}\right)$ which is increasing as n grows, the training regret actually increases for largely randomized methods. In theory, the training regret of RCT is bounded by a constant that does not rely on n when the ϵ-sequence is constant. SRAT-E and SRAT-B perform similarly in terms of regrets on both training and test sets, but SRAT-B has a lower false decision ratio on the training set. The logarithms of their training and test regrets are approximately linear in log⁡n, which is consistent with our theory.

On the test set, AL-GP and RCT perform the best due to their full exploration in the training process. LinUCB needs to fit the regression model of rewards and thus relies on both the main effect and the treatment effect model. In addition, to estimate the upper confidence bound, it needs an assumption on the inference model. With these limitations, the regret or false decision ratio of LinUCB on the test set does not decrease. When n is small, the final ITR estimated by LinUCB can sometimes be optimal since the true ITR is linear. However, the ITR converges to the projection onto that of the linear total reward space when n is large and thus the average regret gets pulled up. On the other hand, OWL tries to find the decision function that maximizes the reward directly. It only requires a correct model of the treatment effect for consistency, without any assumption on the main effect or the distribution of the error term. Therefore, SRATs with $\epsilon_{0}=0.1,\theta =1$ outperform LinUCB on the test set when n is larger than 200.

**Figure 3:**
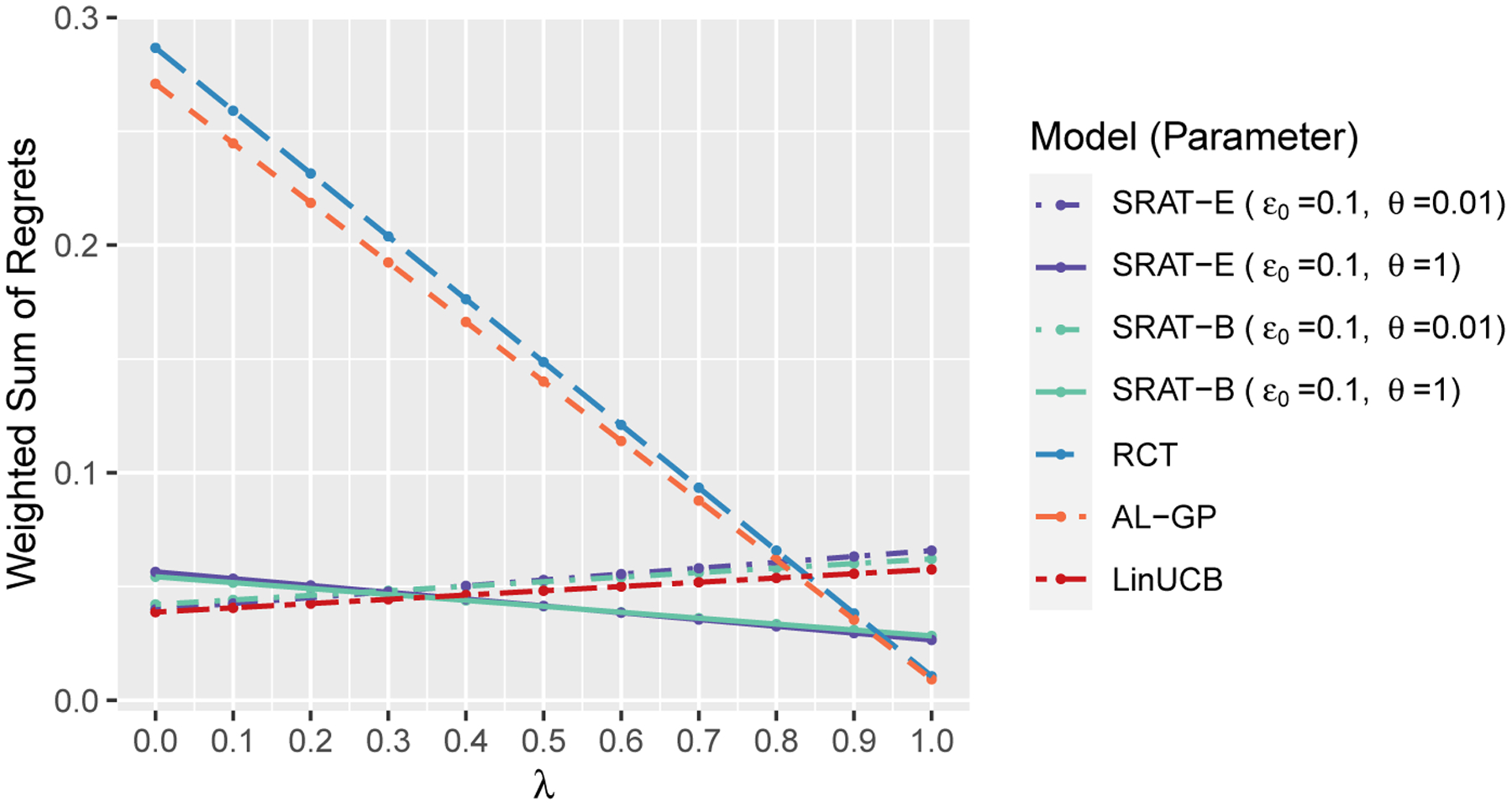
The weighted sum of training and test regrets in scenario 1 when n=800.

We plot a weighted sum of training and test regrets in Figure 3 to show their balance. Specifically, the weighted sum is defined as

$$\lambda {\text{ Regret }}_{\text{test }}+\left(1-\lambda \right){\text{ Regret }}_{\text{train }}=\lambda \frac{1}{n}\overset{n}{\underset{i=1}{\sum }}\left[𝒱\left({\hat{f}}_{i-1}\right)-R_{i}\right]+\left(1-\lambda \right)\left[𝒱\left(f^{\text{*}}\right)-𝒱\left({\hat{f}}_{n}\right)\right]$$

for λ∈[0,1], so that it equals the training regret when λ=0 and equals the test regret when λ=1. The sample size is fixed at 800. The initial value of truncation parameter $\epsilon_{0}$ equals 0.1 and the decay parameter θ takes values in 0.01, 1 for SRAT-E and SRAT-B. The plot shows that we should choose LinUCB when we consider the training regret only, and should choose AL-GP or RCT when we consider the test regret only. However, if we want to consider the performance on both the training and the test sets, we should choose SRAT-E or SRAT-B with θ=1.

**Figure 4:**
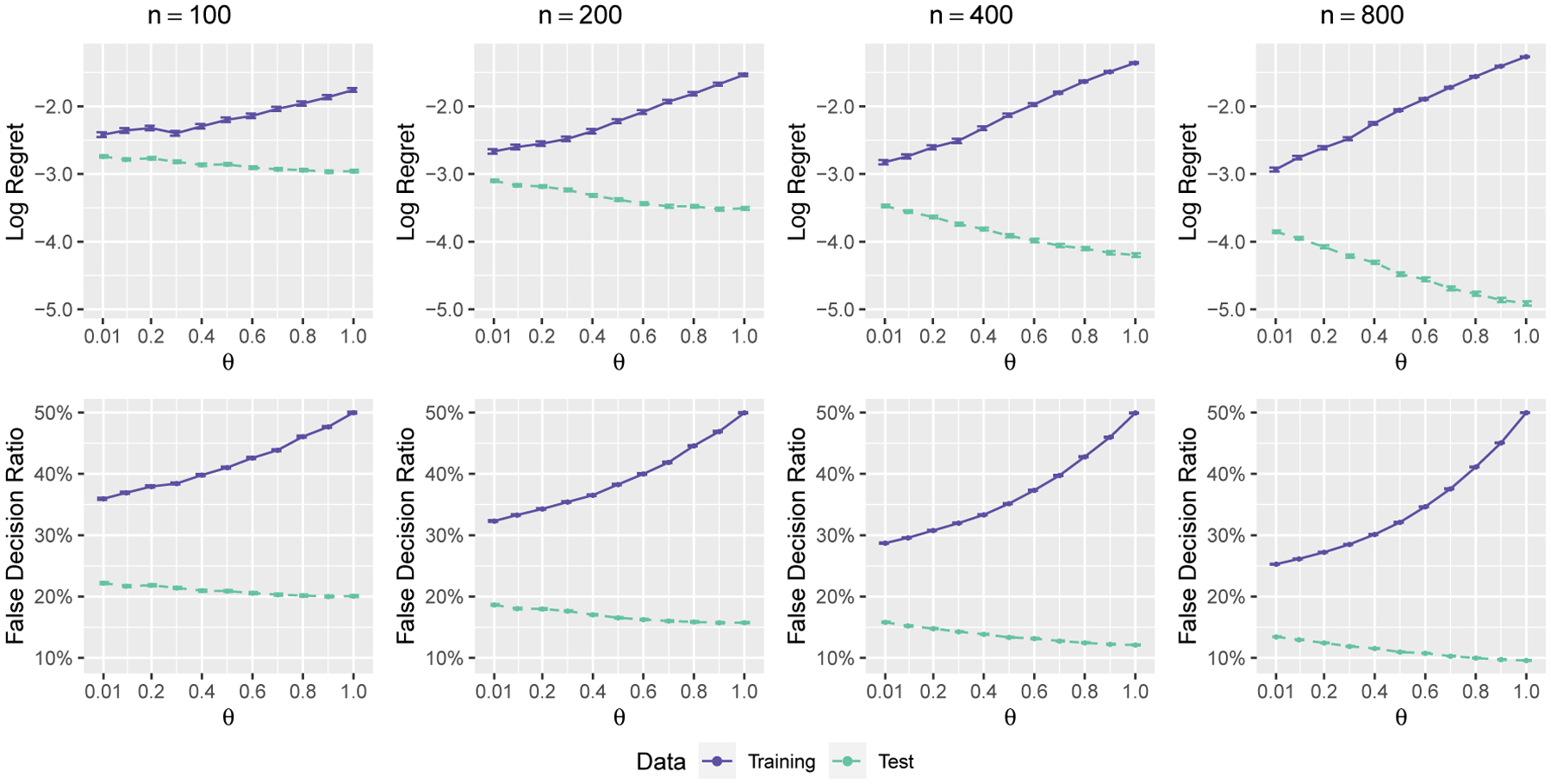
Scenario 1 with $\epsilon_{0}=0.5$. The regret (logarithmic scale) and the false decision ratio on the training or test set against parameter θ.

The change of SRAT-E with different parameters θ and sample size n is demonstrated in Figure 4 for scenario 1. Since SRAT-B performs quite similarly to SRAT-E as shown in Figures 2 and 7, we omit it here to save space. The parameter θ can take values from 0.01, 0.1, 0.2, …, 1 and n can take values from 100, 200, 400, 800. Note that only when $\epsilon_{0}=0.5$ and θ=1, our algorithm represents pure RCT. Thus we only illustrate our findings with $\epsilon_{0}=0.5$ here. Other $\epsilon_{0}$ ‘s give similar conclusion, and smaller $\epsilon_{0}$ means better training performance and worse test performance. The values and standard errors of the mean regret and mean false decision ratio are shown. For all sample sizes, the plots clearly show the tradeoff between training and test performance. Note that when θ increases, $\epsilon_{i}$ increases for all i and the treatments are more randomized in the training process. While the training regret increases with more randomization, the test regret decreases. The false decision ratio shows a similar tendency. All the points with θ=1 have an accuracy of 50% on the training set, which indeed illustrates the pure randomization. In accordance with the theory, the logarithm of training and test regrets are approximately linear in θ. In practice, the training regret is more affected than the test regret by θ. As shown in Figure 4, when n=800, the training regret increases by $e^{-1.27}-e^{-2.93}=0.227$ while the test regret decreases by $e^{-3.85}-e^{-4.91}=0.014$ when θ increases from 0.01 to 1.

**Figure 5:**
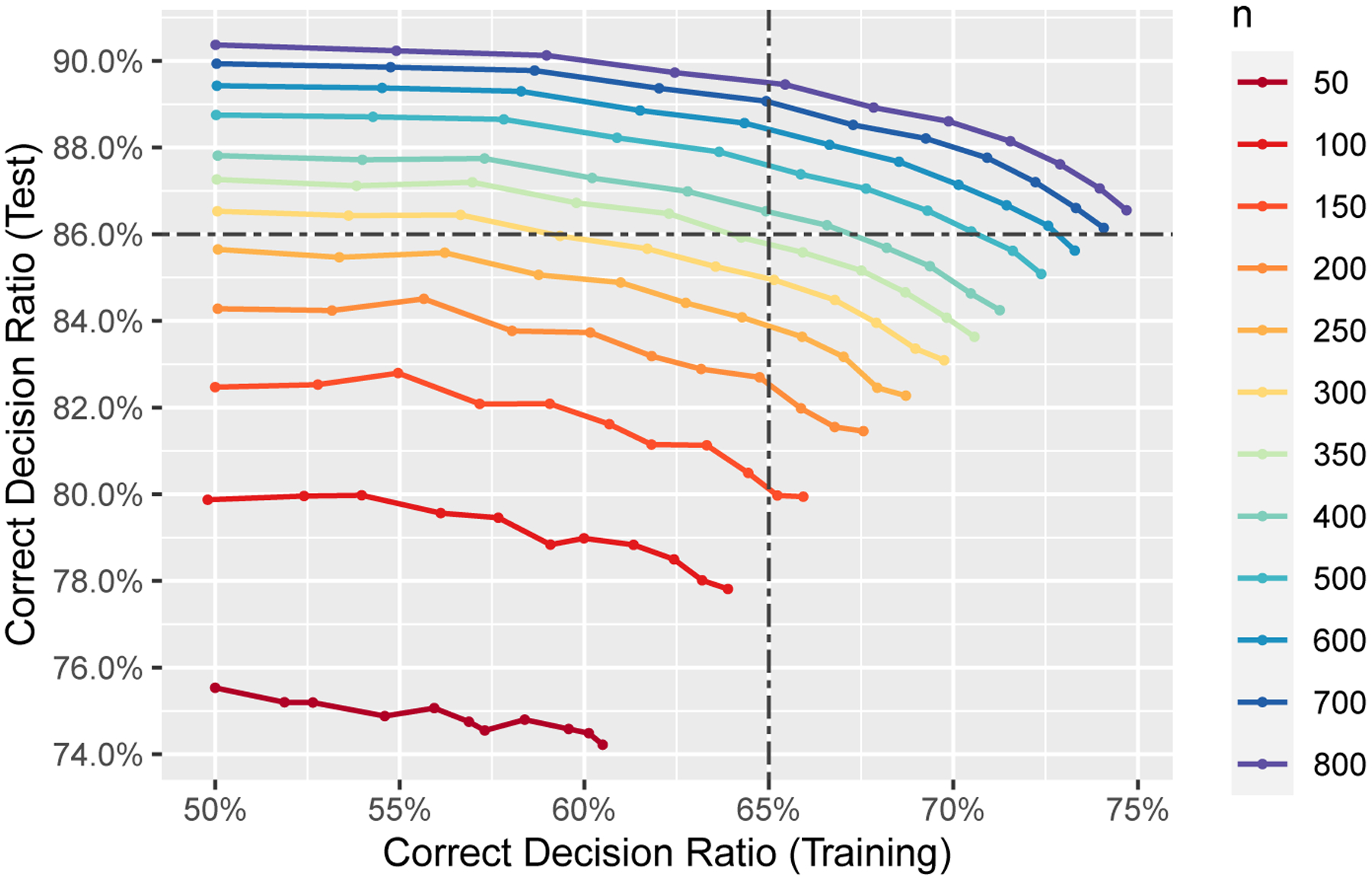
Sample size consideration for SRAT-E in scenario 1 with $\epsilon_{0}=0.5$. Correct decision ratios on the test set against that on the training set. Each line represents a sample size n and each point on the line represents a value of θ. Points to the right correspond to smaller θ, and thus lead to higher correct decision ratio on the training set and lower ratio on the test set.

Using this simulation example, we can also illustrate how to find the sample size needed for a clinical trial of certain purposes. Given different requirements for the trial and the population, we need different sample sizes. Here we illustrate the situation when the proportion of patients assigned the better treatments is required to reach a certain level in Figure 5 for SRAT-E in scenario 1. Note that the variation trends of correct decision ratios against θ are opposite for the training and test data. In particular, θ should be small enough so that the decision process is greedy on the training set, and in the meanwhile it should be large enough so that the final ITR is efficient on the test set. It is clear that the two accuracies are negatively correlated. For example, when we need the training ratio to be greater than 65%, θ≤0.1 for n=150,θ≤0.2 for n=200,θ≤0.3 for n=250,θ≤0.4 for n=300,θ≤0.4 for n=350,θ≤0.4 for n=400,θ≤0.5 for n=500,θ≤0.5 for n=600,θ≤0.5 for n=700, and θ≤0.6 for n=800 will all do. When we need the test ratio to be greater than 86%,θ≥0.8 for n=300,θ≥0.6 for n=350,θ≥0.4 for n=400,θ≥0.2 for n=500,θ≥0.1 for n=600, any θ for n=700, and any θ for n=800 all satisfy the requirement. However, only points lie in the top right rectangle marked by the two dot-dashed lines meet the two requirements simultaneously. The smallest sample size among these points is n=400, with θ=0.4. Other levels of the correct decision ratios and their required sample sizes are listed in Table 1. Since larger θ generates better ITR and ITR is our ultimate goal, we report the largest θ corresponding to the minimum sample size required.

## 6. Real Data Analysis

We use a real study to illustrate the performance of the proposed method. The Nefazodone-CBASP trial was designed to compare the efficacy of several treatment options for patients with nonpsychotic chronic major depressive disorder (MDD) (Keller et al., 2000). Specifically, 681 outpatients were randomized to either Nefazodone, Cognitive Behavioral-Analysis System of Psychotherapy (CBASP), or the combination of Nefazodone and CBASP with equal probabilities. The primary outcome was the score on the 24-item Hamilton Rating Scale for Depression (HRSD). Lower HRSD scores indicate satisfactory therapeutic efficacy. T-tests have shown that the combination treatment generated significantly lower HRSD scores than the other two treatments, and there are no significant differences between the Nefazodone group and the CBASP group. However, CBASP requires two onsite visits to the clinic weekly, which burdens patients compared with Nefazodone alone. Consequently, we want to investigate whether CBASP is necessary for all patients. Here we compare Nefazodone with the combination treatment only. We consider three feature variables for treatment suggestions: the baseline HRSD scores, the alcohol dependence, and the HAMA somatic anxiety scores, following Minsker et al. (2016), which referred to Gunter et al. (2007). There were 436 patients with complete information on treatments, rewards and feature variables, among which 216 were randomized to Nefazodone and 220 belonged to the combined treatment group.

To simulate an adaptive clinical trial, we first generate a treatment suggestion based on the tailoring variables of the next patient using our algorithm. If the actual treatment taken is consistent with our suggestion, we take down the whole record of this patient, including feature variables, the treatment and the reward; otherwise, we drop this record and move on to the next. Note that the first $n_{0}$ suggestions are given with equal probabilities on each treatment. Five-fold cross validation is used here to avoid overfitting. Specifically, the data set is partitioned into five parts randomly. Four of the five parts are used iteratively as training data to apply our algorithm in generating the treatment suggestion. The last part is used as the test set to evaluate the ITR. The performance on the test data is evaluated using an unbiased estimator of the value function 𝒱(f) (Qian and Murphy, 2011; Minsker et al., 2016)

$$\overset{n}{\underset{i=1}{\sum }}\frac{R_{i}1\left[A_{i}=\text{sign}\left\{f\left(X_{i}\right)\right\}\right]}{\pi_{i}\left(A_{i};X_{i}\right)}/\overset{n}{\underset{i=1}{\sum }}\frac{1\left[A_{i}=\text{sign}\left\{f\left(X_{i}\right)\right\}\right]}{\pi_{i}\left(A_{i};X_{i}\right)}.$$

Here the rewards $R_{i}$ ‘s are defined as the negative HRSD scores.

**Figure 6:**
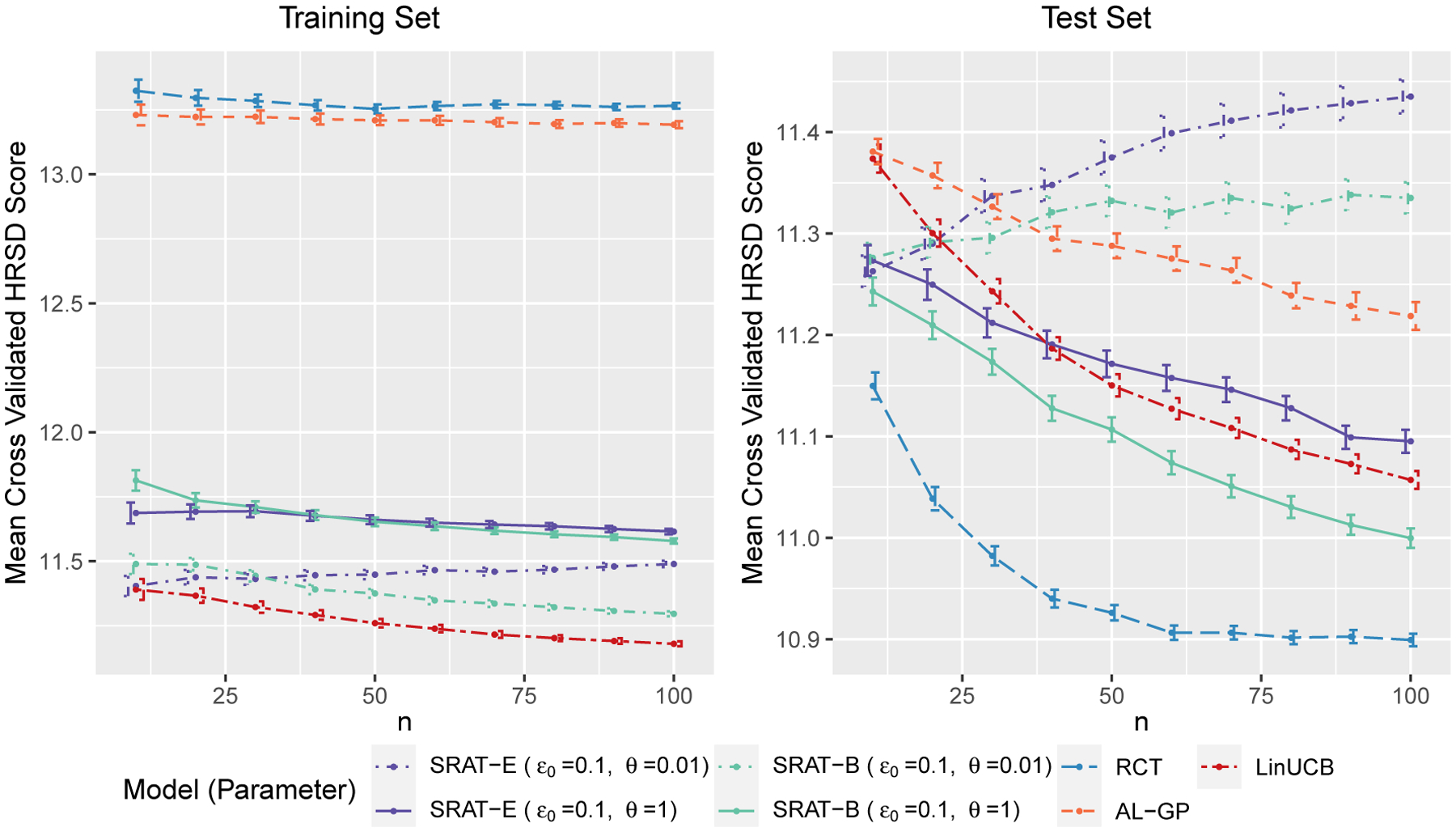
Mean cross-validated HRSD scores against the sample size n.

**Figure 7:**
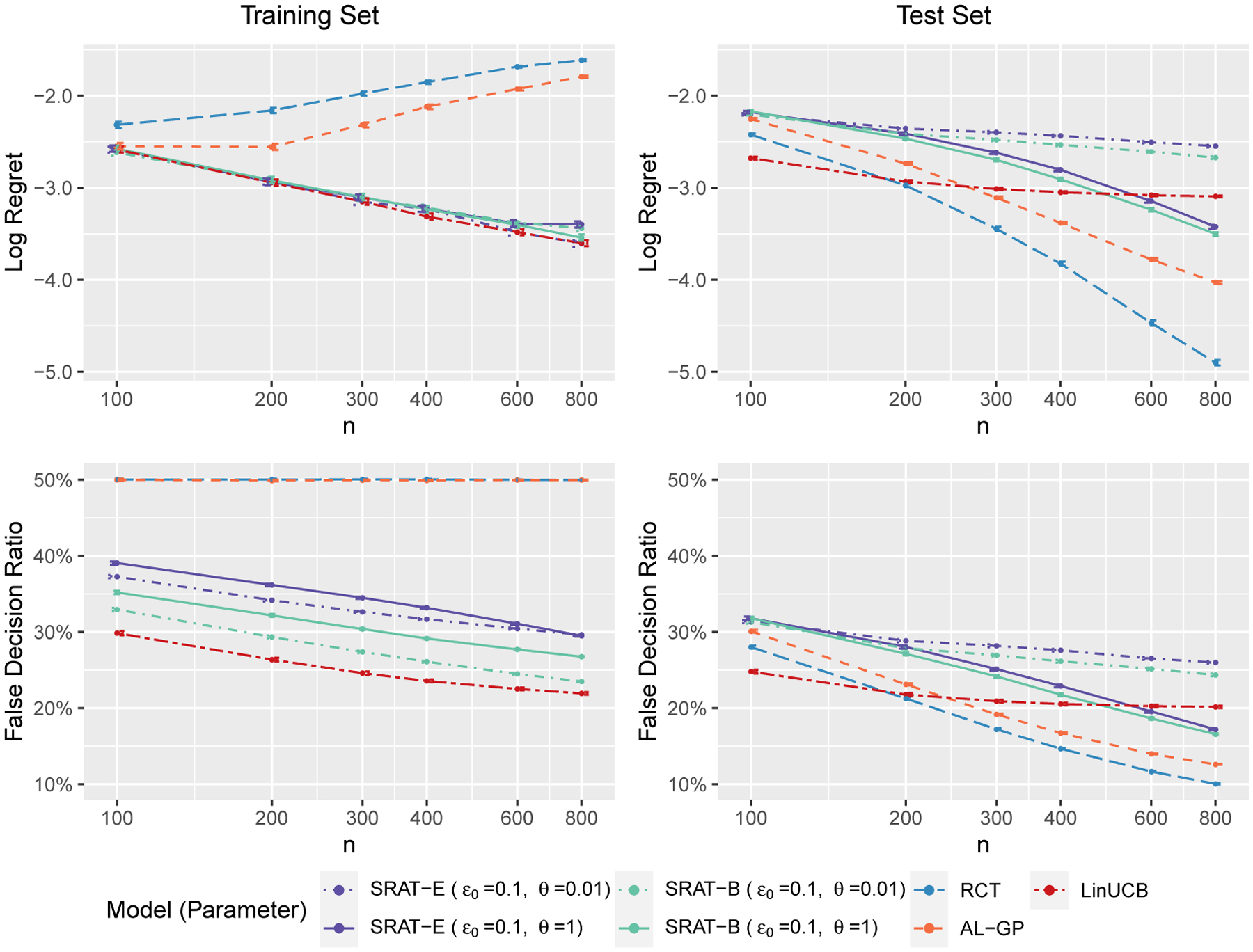
Scenario 2. The regret (logarithmic scale) and the false decision ratio on the training or test set against sample size n.

The initial sample size $n_{0}$ is fixed at 50. The recruitment stops when the sample size n reaches 100, or the training data run out. We average the mean reward on each test fold for n=10,20,…,100. The process is repeated 1,000 times. Finally, the means and standard errors of means across all iterations are reported. From Section 5, we know that the training and test values are monotone in $\epsilon_{0}$ and θ. Therefore, we only demonstrate the situation when $\epsilon_{0}=0.1$ and θ=0.01,1. The contextual bandit algorithm LinUCB and the active clinical trial method AL-GP are also compared here. Figure 6 displays the negative mean rewards, that is, the mean cross validated HRSD scores, against the sample size n. Lower scores are more satisfactory.

On the training set, LinUCB produces the least HRSD scores on the training set and SRAT-B with ϵ=0.1,θ=0.01 is the second best. Note that LinUCB can be viewed as a limiting case of SRAT-B on the training set as discussed before, and is actually the greediest among the algorithm family. Patients taking purely randomized treatments suggested by RCT and AL-GP have higher HRSD scores. On the test set, RCT produces the most desirable HRSD score, followed by SRAT-B with θ=1. LinUCB is slightly worse due to its greediness. AL-GP is not competitive on both sets, maybe because the nonparametric method is not efficient when the sample size is small.

## 7. Discussion

Our goal is to construct an efficient ITR, and in the meantime make the data collection process, the clinical trial, as beneficial to the patients as possible. We propose a classification-based bandit algorithm, SRAT, that uses OWL to update the ITR and ϵ-greedy or a variation of Boltzmann exploration for exploration. This is a work of finding the tradeoff between the ethics of patients involved in the clinical trial and the general population. We also present a new theoretical analysis tool based on empirical process for estimating finite sample risk bound on martingale sequences. Given different requirements of training and test performance, the sample size needed is illustrated by simulation.

In this paper, we assume that the true optimal decision function lies in the function class where we search for the estimated function, and proved a $n^{-1/2}$ convergence rate of test regret up to logarithmic factors for a constant ${\left\{\epsilon_{i}\right\}}_{i=1}^{n}$ sequence. If tailoring variables have high dimensions, a penalty term can be added in finding the optimal solution to avoid overfitting. For i.i.d. data, when Gaussian kernel is used with a penalty term and the optimal function need not be in the function class, Steinwart and Scovel (2007) proved a rate faster than $n^{-1/2}$ for SVM under Tsybakov’s noise assumption and geometric noise assumption, and Zhao et al. (2012) proved a rate a little bit slower than $n^{-1/2}$ for OWL under the geometric noise assumption. How to extent these ideas to sequentially generated data is still an open question.

Currently, the estimated ITR is updated after each trial. However, it can be a burden on the computation resources and running time if the algorithm runs slowly or the sample size is too large. Batch sampling is an efficient approach that worths investigation. Apart from accelerating the training process, it also allows in-time evaluation of the current estimated optimal ITR. Part of the batch can be drew randomly as a test set. How the estimation improves through time can be recorded as well.

Another interesting question is how to set up an early stopping rule. We can stop enrolling new patients into a clinical trial if the learnt ITR is good enough. This can be done by constructing a confidence interval for the estimated value of the learnt ITR. If we have enough confidence that the estimated value is satisfactory in the clinical sense, we can stop the trial at this point. Future work is needed on constructing a confidence interval for sequentially generated data.

This article focuses on a single-stage problem. However, it is widely recognized that some diseases require multiple treatments throughout the therapeutic session. For example, the sequential multiple assignment randomized trial (SMART) is a way of connecting potential outcomes with observed data (Lavori and Dawson, 2000; Murphy, 2005; Murphy et al., 2007). Patients are randomized at every decision point. An abundance of literature has discussed this issue on independent data (Zhao et al., 2015; Liu et al., 2018). Problems on infinite horizon can be solved with additional Markovian assumptions and offline data (Luckett et al., 2020). However, multi-stage decision problems with slack constraints on the value function or with online data still worth investigation.

## Supplementary Material

1

## Figures and Tables

**Table 1:** Clinical trial sample sizes needed for different requirements of correct decisionratios on the training and test sets.

Training	Test				
	0.74	0.78	0.82	0.86	0.90
0.49	50(1.0)	100(1.0)	150(1.0)	300(1.0)	800(1.0)
0.55	50(0.6)	100(0.7)	150(0.7)	300(0.8)	800(0.8)
0.60	50(0.1)	100(0.3)	200(0.6)	350(0.6)	
0.65	150(0.1)	150(0.1)	250(0.3)	400(0.4)	
0.70	350(0.01)	350(0.01)	350(0.01)	500(0.2)	

## References

[R1] Auer Peter. Using confidence bounds for exploitation-exploration trade-offs. Journal of Machine Learning Research, 3(Nov):397–422, 2002.

[R2] Bae Jongsig and Levental Shlomo. Uniform CLT for Markov chains and its invariance principle: a martingale approach. Journal of Theoretical Probability, 8(3):549–570, 1995.

[R3] Bartlett Peter L, Jordan Michael I, and McAuliffe Jon D. Convexity, classification, and risk bounds. Journal of the American Statistical Association, 101(473):138–156, 2006.

[R4] Bastani Hamsa and Bayati Mohsen. Online decision making with high-dimensional covariates. Operations Research, 68(1):276–294, 2020.

[R5] Bousquet Olivier. A Bennett concentration inequality and its application to suprema of empirical processes. Comptes Rendus Mathematique, 334(6):495–500, 2002.

[R6] Bubeck Sébastien, Munos Rémi, and Stoltz Gilles. Pure exploration in multi-armed bandits problems. In International Conference on Algorithmic Learning Theory, pages 23–37. Springer, 2009.

[R7] Chambaz Antoine, Zheng Wenjing, and van der Laan Mark J. Targeted sequential design for targeted learning inference of the optimal treatment rule and its mean reward. Annals of Statistics, 45(6):2537, 2017. 29398733 10.1214/16-AOS1534 PMC5794253

[R8] Chapelle Olivier and Li Lihong. An empirical evaluation of Thompson sampling. In Advances in Neural Information Processing Systems, pages 2249–2257, 2011.

[R9] Chapple Andrew G and Thall Peter F. A hybrid phase I-II/III clinical trial design allowing dose re-optimization in phase III. Biometrics, 75(2):371–381, 2019. 30367457 10.1111/biom.12994 PMC6486466

[R10] Chen Haoyu, Lu Wenbin, and Song Rui. Statistical inference for online decision making: In a contextual bandit setting. Journal of the American Statistical Association, pages 1–16, 2020. 10.1080/01621459.2020.1770098 PMC7962379 33737759

[R11] Chen Jingxiang, Fu Haoda, He Xuanyao, Michael R Kosorok, and Yufeng Liu. Estimating individualized treatment rules for ordinal treatments. Biometrics, 74(3):924–933, 2018. 29534296 10.1111/biom.12865 PMC6136994

[R12] Chen Yuan, Liu Ying, Zeng Donglin, and Wang Yuanjia. DTRlearn2: Statistical Learning Methods for Optimizing Dynamic Treatment Regimes, 2019. URL https://CRAN.R-project.org/package=DTRlearn2.. R package version 1.0

[R13] Chow Shein-Chung. Adaptive clinical trial design. Annual Review of Medicine, 65:405–415, 2014. 10.1146/annurev-med-092012-112310 24422576

[R14] Chu Wei, Li Lihong, Reyzin Lev, and Schapire Robert. Contextual bandits with linear payoff functions. In International Conference on Artificial Intelligence and Statistics, pages 208–214, 2011.

[R15] Freedman David A. On tail probabilities for martingales. Annals of Probability, pages 100–118, 1975.

[R16] Gunter Lacey, Zhu Ji, and Murphy Susan. Variable selection for optimal decision making. In Conference on Artificial Intelligence in Medicine in Europe, pages 149–154. Springer, 2007.

[R17] Hamburg Margaret A and Collins Francis S. The path to personalized medicine. New England Journal of Medicine, 363(4):301–304, 2010. 20551152 10.1056/NEJMp1006304

[R18] Hu Feifang and Rosenberger William F. The Theory of Response-Adaptive Randomization in Clinical Trials. John Wiley & Sons, 2006.

[R19] Hu Jianhua, Zhu Hongjian, and Hu Feifang. A unified family of covariate-adjusted response-adaptive designs based on efficiency and ethics. Journal of the American Statistical Association, 110(509):357–367, 2015. 26120220 10.1080/01621459.2014.903846 PMC4478080

[R20] Keller Martin B, McCullough James P, Klein Daniel N, Arnow Bruce, Dunner David L, Gelenberg Alan J, Markowitz John C, Nemeroff Charles B, Russell James M, Thase Michael E, A comparison of nefazodone, the cognitive behavioral-analysis system of psychotherapy, and their combination for the treatment of chronic depression. New England Journal of Medicine, 342(20):1462–1470, 2000. 10816183 10.1056/NEJM200005183422001

[R21] Kim Edward S, Herbst Roy S, Wistuba Ignacio I, Lee J Jack, Blumenschein George R, Tsao Anne, Stewart David J, Hicks Marshall E, Erasmus Jeremy, Gupta Sanjay, The BATTLE trial: personalizing therapy for lung cancer. Cancer Discovery, 1(1):44–53, 2011. 22586319 10.1158/2159-8274.CD-10-0010 PMC4211116

[R22] Krause Andreas and Ong Cheng S. Contextual Gaussian process bandit optimization. In Advances in Neural Information Processing Systems, pages 2447–2455, 2011.

[R23] Lai Tze Leung, Lavori Philip W, Shih Mei-Chiung I, and Sikic Branimir I. Clinical trial designs for testing biomarker-based personalized therapies. Clinical Trials, 9(2):141–154, 2012. 22397801 10.1177/1740774512437252 PMC4296980

[R24] Lattimore Tor and Szepesvári Csaba. Bandit Algorithms. Cambridge University Press, 2020.

[R25] Lavori Philip W and Dawson Ree. A design for testing clinical strategies: biased adaptive within-subject randomization. Journal of the Royal Statistical Society. Series A, 163(1): 29–38, 2000.

[R26] Li Lihong, Chu Wei, Langford John, and Schapire Robert E. A contextual-bandit approach to personalized news article recommendation. In International Conference on World Wide Web, pages 661–670, 2010.

[R27] Liao Peng, Greenewald Kristjan, Klasnja Predrag, and Murphy Susan. Personalized heart-steps: A reinforcement learning algorithm for optimizing physical activity. Proceedings of the ACM on Interactive, Mobile, Wearable and Ubiquitous Technologies, 4(1):1–22, 2020. 34527853 10.1145/3381007 PMC8439432

[R28] Liu Ying, Wang Yuanjia, Michael R Kosorok Yingqi Zhao, and Zeng Donglin. Augmented outcome-weighted learning for estimating optimal dynamic treatment regimens. Statistics in Medicine, 37(26):3776–3788, 2018. 29873099 10.1002/sim.7844 PMC6191367

[R29] Luckett Daniel J, Laber Eric B, Kahkoska Anna R, Maahs David M, Mayer-Davis Elizabeth, and Kosorok Michael R. Estimating dynamic treatment regimes in mobile health using v-learning. Journal of the American Statistical Association, 115(530):692–706, 2020. 32952236 10.1080/01621459.2018.1537919 PMC7500510

[R30] Minsker Stanislav, Zhao Ying-Qi, and Cheng Guang. Active clinical trials for personalized medicine. Journal of the American Statistical Association, 111(514):875–887, 2016. 28018014 10.1080/01621459.2015.1066682 PMC5179145

[R31] Murphy Susan A. An experimental design for the development of adaptive treatment strategies. Statistics in Medicine, 24(10):1455–1481, 2005. 15586395 10.1002/sim.2022

[R32] Murphy Susan A, Oslin David W, Rush A John, and Zhu Ji. Methodological challenges in constructing effective treatment sequences for chronic psychiatric disorders. Neuropsychopharmacology, 32(2):257–262, 2007. 17091129 10.1038/sj.npp.1301241

[R33] Nishiyama Yoichi. Some central limit theorems for ℓ_infty_-valued semimartingales and their applications. Probability Theory and Related Fields, 108(4):459–494, 1997.

[R34] Nishiyama Yoichi Weak convergence of some classes of martingales with jumps. Annals of Probability, 28(2):685–712, 2000.

[R35] Perchet Vianney and Rigollet Philippe. The multi-armed bandit problem with covariates. Annals of Statistics, 41(2):693–721, 2013.

[R36] Qian Min and Murphy Susan A. Performance guarantees for individualized treatment rules. Annals of Statistics, 39(2):1180, 2011. 21666835 10.1214/10-AOS864 PMC3110016

[R37] Rakhlin Alexander and Sridharan Karthik. Statistical learning and sequential prediction. Book Draft, 2014.

[R38] Rakhlin Alexander, Sridharan Karthik, and Tewari Ambuj. Sequential complexities and uniform martingale laws of large numbers. Probability Theory and Related Fields, 161 (1–2):111–153, 2015.

[R39] Renfro Lindsay A, Mallick Himel, An Ming-Wen, Sargent Daniel J, and Mandrekar Sumithra J. Clinical trial designs incorporating predictive biomarkers. Cancer Treatment Reviews, 43:74–82, 2016. 26827695 10.1016/j.ctrv.2015.12.008 PMC4737867

[R40] Riviere Marie-Karelle, Yuan Ying, Jourdan Jacques-Henri, Dubois Frédéric, and Zohar Sarah. Phase I/II dose-finding design for molecularly targeted agent: plateau determination using adaptive randomization. Statistical Methods in Medical Research, 27(2):466–479, 2018. 26988926 10.1177/0962280216631763

[R41] Steinwart Ingo and Scovel Clint. Fast rates for support vector machines using Gaussian kernels. Annals of Statistics, 35(2):575–607, 2007.

[R42] Sutton Richard S and Barto Andrew G. Reinforcement Learning: An Introduction. MIT press, 2018.

[R43] Talagrand Michel. Sharper bounds for Gaussian and empirical processes. Annals of Probability, pages 28–76, 1994.

[R44] Tewari Ambuj and Murphy Susan A. From ads to interventions: Contextual bandits in mobile health. In Mobile Health, pages 495–517. Springer, 2017.

[R45] Thall Peter F. Ethical issues in oncology biostatistics. Statistical methods in medical research, 11(5):429–448, 2002. 12357588 10.1191/0962280202sm301ra

[R46] Thall Peter F, Nguyen Hoang Q, Braun Thomas M, and Qazilbash Muzaffar H. Using joint utilities of the times to response and toxicity to adaptively optimize schedule-dose regimes. Biometrics, 69(3):673–682, 2013. 23957592 10.1111/biom.12065 PMC3963428

[R47] Van de Geer Sara. Exponential inequalities for martingales, with application to maximum likelihood estimation for counting processes. Annals of Statistics, pages 1779–1801, 1995.

[R48] Van der Vaart Aad W and Wellner Jon A. Weak Convergence and Empirical Processes: With Applications to Statistics. Springer, 1996.

[R49] Yang Yuhong and Zhu Dan. Randomized allocation with nonparametric estimation for a multi-armed bandit problem with covariates. Annals of Statistics, 30(1):100–121, 2002.

[R50] Zhang Li-Xin, Hu Feifang, Siu Hung Cheung, and Wai Sum Chan. Asymptotic properties of covariate-adjusted response-adaptive designs. Annals of Statistics, 35(3):1166–1182, 2007.

[R51] Zhao Ying-Qi, Zeng Donglin, Eric B Laber, and Michael R Kosorok. New statistical learning methods for estimating optimal dynamic treatment regimes. Journal of the American Statistical Association, 110(510):583–598, 2015. 26236062 10.1080/01621459.2014.937488 PMC4517946

[R52] Zhao Yingqi, Zeng Donglin, A John Rush, and Michael R Kosorok. Estimating individualized treatment rules using outcome weighted learning. Journal of the American Statistical Association, 107(499):1106–1118, 2012. 23630406 10.1080/01621459.2012.695674 PMC3636816

[R53] Zhou Dongruo, Li Lihong, and Gu Quanquan. Neural contextual bandits with UCB-based exploration. In International Conference on Machine Learning, pages 11492–11502. PMLR, 2020.

[R54] Zhou Xin, Nicole Mayer-Hamblett Umer Khan, and Kosorok Michael R. Residual weighted learning for estimating individualized treatment rules. Journal of the American Statistical Association, 112(517):169–187, 2017. 28943682 10.1080/01621459.2015.1093947 PMC5607057

