# Supplementary material for: Non-asymptotic Properties of Individualized Treatment Rules from Sequentially Rule-Adaptive Trials
Source: J Mach Learn Res. Author manuscript; Available in PMC 2023 Aug 11. (PMC10419117)
Supplement: 1 [file NIHMS1918139-supplement-1.pdf]

Appendix A. Preliminaries

We provide useful lemmas used for proving our main theorems, among which Lemmas 12 to 15 are quoted from existing literature without proof.

Talagrand's inequality (Talagrand, 1994; Bousquet, 2002) below is used to prove the convergence rate in Theorem 1 for i.i.d. data in the pilot trial. The following version is taken from Steinwart and Scovel (2007, Theorem 5.3).

Lemma 12 (Talagrand's inequality) *Assume* $\{\boldsymbol{X}_i\}_{i=1}^n$ *are independent* $\mathcal{X}$*-valued random variables on* $\mathbb{P}$*. Let* $\mathcal{F}$ *be a countable set of functions from* $\mathcal{X}$ *to* $\mathbb{R}$ *and assume that all functions* f *in* $\mathcal{F}$ *are* $\mathbb{P}$*-measurable, square-integrable such that* $\|f\|_\infty \leq U < \infty$ *and* $\mathbb{E}[f(\boldsymbol{X}_1)] = \cdots = \mathbb{E}[f(\boldsymbol{X}_n)] = 0$*. Let* $Z := \sup_{f\in\mathcal{F}} \sum_{i=1}^n f(\boldsymbol{X}_i)$*,* $\mu^* := \mathbb{E}Z$ *and let* σ^2 *be a positive real number such that* $\sigma^2 \geq \frac{1}{n}\sum_{i=1}^n \sup_{f\in\mathcal{F}} \mathrm{Var}[f(\boldsymbol{X}_i)]$*. Then for all* $\delta \geq 0$*,*

$$\mathbb{P}\left(Z \geq 3\mu^* + \sqrt{2\delta\sigma^2 n} + U\delta\right) \leq e^{-\delta}.$$

The following lemma gives an analogy of Talagrand's inequality on martingale processes. A $\mathcal{Z}$-valued tree $\boldsymbol{z}$ of depth n is a rooted complete binary tree with nodes generated by elements of $\mathcal{Z}$. The tree $\boldsymbol{z} = (\boldsymbol{z}_1, \ldots, \boldsymbol{z}_n)$ is a sequence of labeling functions such that $\boldsymbol{z}_i : \{\pm 1\}^{i-1} \mapsto \mathcal{Z}$. Let $\boldsymbol{\eta} = (\eta_1, \ldots, \eta_n)$ be a sequence of i.i.d. Rademacher random variables. Then the sequential Rademacher complexity of a function class $\mathcal{F} \subset \mathbb{R}^{\mathcal{Z}}$ on a $\mathcal{Z}$-valued tree $\boldsymbol{z}$ is defined as

$$\mathcal{R}_n(\mathcal{F}, \boldsymbol{z}) := \mathbb{E}\left[\sup_{f\in\mathcal{F}} \frac{1}{n}\sum_{i=1}^n \eta_i f(\boldsymbol{z}_i(\boldsymbol{\eta}))\right].$$

Further, define

$$\mathcal{R}_n(\mathcal{F}) := \sup_{\boldsymbol{z}} \mathcal{R}_n(\mathcal{F}, \boldsymbol{z}),$$

and Rakhlin et al. (2015) showed that

$$\frac{1}{2}\mathbb{E}\sup_{f\in\mathcal{F}} \mathbb{M}_n(f) \leq \mathcal{R}_n(\mathcal{F}) \leq 2\sup_{\mathbb{P}} \mathbb{E}\sup_{f\in\mathcal{F}} \mathbb{M}_n(f) + \frac{D}{2\sqrt{n}}, \tag{8}$$

where $D = \inf_{z\in\mathcal{Z}} \sup_{f,f'\in\mathcal{F}}[f(z) - f'(z)] \geq 0$. This indicates that $\mathcal{R}_n(\mathcal{F})$ and the expectation of the martingale process suprema $\sup_{\mathbb{P}} \mathbb{E} \sup_{f\in\mathcal{F}} \mathbb{M}_n(f)$ are on the same scale.

The covering numbers are also extended to sequential data. A set V of $\mathbb{R}$-valued trees of depth n is a (sequential) ϵ-cover with respect to L_p-norm of $\mathcal{F} \subset \mathbb{R}^{\mathcal{Z}}$ on a tree z of depth n if for any $f \in \mathcal{F}$ and any $\eta \in \{\pm 1\}^n$, there exists $\boldsymbol{v} \in V$ such that $\left(\frac{1}{n}\sum_{i=1}^n |\boldsymbol{v}_i(\eta) - f(\boldsymbol{z}_i(\eta))|^p\right)^{1/p} \leq \epsilon$. The sequential covering number of a function class $\mathcal{F}$ on a given tree $\boldsymbol{z}$ is defined as

$$\mathcal{N}_p(\epsilon, \mathcal{F}, \boldsymbol{z}) = \min\left\{|V| : \text{ V is an } \epsilon\text{-cover with respect to } L_p\text{-norm of } \mathcal{F} \text{ on } \boldsymbol{z}\right\}.$$

Moreover, define the maximal L_p covering number of $\mathcal{F}$ over depth-n trees as $\mathcal{N}_p(\epsilon, \mathcal{F}, n) = \sup_{\boldsymbol{z}} \mathcal{N}_p(\epsilon, \mathcal{F}, \boldsymbol{z})$.

Lemma 13 (Lemma 15 in Rakhlin et al. 2015) *For $\mathcal{F} \subset [-1,1]^{\mathcal{Z}}$, for $n \geq 2$ and any $t > 0$, we have that*

$$\mathbb{P}\left(\sup_{f\in\mathcal{F}}\left|\frac{1}{n}\sum_{i=1}^n f(Z_i) - \mathbb{E}_{i-1}f(Z_i)\right| > t\right) \leq 8L\exp\left(-\frac{t^2}{c\log^3 n\mathcal{R}_n^2(\mathcal{F})}\right) \tag{9}$$

under the mild assumptions $\mathcal{R}_n(\mathcal{F}) \geq 1/n$ and $\mathcal{N}_\infty(2^{-1}, \mathcal{F}, n) \geq 4$. Here c is an absolute constant and $L > e^4$ is such that $L > \sum_{j=1}^\infty \mathcal{N}_\infty(2^{-j}, \mathcal{F}, n)^{-1}$.

From the above lemma, we can see that the concentration inequality essentially relies on the sequential Rademacher complexity $\mathcal{R}_n(\mathcal{F})$, which can be upper and lower bounded by functions of $\mathbb{E}\sup_{f\in\mathcal{F}} \mathbb{M}_n(f)$.

To obtain a bound on the suprema of martingale process over a finite set, we take use of a martingale inequality and a conclusion about L_ψ-Orlicz norm. Freedman's inequality is an extension of Bernstein's inequality to martingale difference sequences.

Lemma 14 (Freedman's inequality, Freedman 1975) *Suppose $\{X_i\}_{i\geq 1}$ is a $\mathcal{G}_i$-adapted martingale difference sequence and $S_n = \sum_{i=1}^n X_i$. Then for all $t > 0$,*

$$\mathbb{P}(S_n \geq t) \leq \exp\left\{-\frac{1}{2}\frac{t^2}{\|\langle S\rangle_n\|_\infty + \max_i \|X_i\|_\infty t/3}\right\},$$

where $\langle S\rangle_n = \sum_{i=1}^n \mathbb{E}\left(X_i^2|\mathcal{G}_{i-1}\right)$ is the quadratic variation of S.

The following lemma gives a bound on the expectation of suprema over a finite set using L_ψ-Orlicz norm.

Lemma 15 (Van der Vaart and Wellner 1996) *Suppose that $X_1, \ldots, X_n$ are arbitrary random variables satisfying the probability tail bound*

$$\mathbb{P}(|X_i| > t) \leq 2\exp\left\{-\frac{1}{2}\frac{t^2}{d + cx}\right\},$$

for all $t > 0$ and $i = 1, \ldots, n$ for fixed positive numbers c and d. Then there is a universal $K < \infty$ so that

$$\left\| \max_{1 \le i \le n} |X_i| \right\|_{\psi_1} \le K \left\{ c \operatorname{Log} n + \sqrt{d}\sqrt{\operatorname{Log} n} \right\},$$

where the L_ψ-Orlicz norm is defined as $\|X\|_\psi = \inf\{c > 0 : \mathbb{E}\psi(|X|/c) \le 1\}$ for any random variable X, and $\psi_p = e^{x^p} - 1$ is a Young modulus for each $p \ge 1$.

The following inequality is a key step in the proof of Lemma 6. It bounds the expectation of the suprema of a martingale process over a finite set, after which the bound on a general set can be derived.

Corollary 16 *Suppose $\{X_i\}_{i \ge 1}$ is a $\mathcal{X}$-valued, $\mathcal{G}_i$-adapted martingale difference sequence. For any finite set $\mathcal{F} : \mathcal{X} \mapsto \mathbb{R}$,*

$$\mathbb{E}\left\|\sqrt{n}\mathbb{M}_n\right\|_{\mathcal{F}} \lesssim \frac{1}{\sqrt{n}} \max_{f \in \mathcal{F}} \|f\|_\infty \operatorname{Log}|\mathcal{F}| + \frac{1}{\sqrt{n}} \max_{f \in \mathcal{F}} \sqrt{\|\langle M \rangle_n\|_\infty} \sqrt{\operatorname{Log}|\mathcal{F}|},$$

where $\langle M \rangle_n = \sum_{i=1}^n \operatorname{Var}[f(X_i)|\mathcal{G}_{i-1}]$.

Proof First we rewrite Lemma 14 in the form of a martingale process. For a $\mathcal{G}_i$-adapted sequence $\{Y_i\}_{i \ge 1}$, take $\mathbb{E}[f(Y_i)|\mathcal{G}_{i-1}] - f(Y_i)$ as X_i in Lemma 14, which is a martingale difference sequence. The supremum term can be bounded as $\|\mathbb{E}[f(Y_i)|\mathcal{G}_{i-1}] - f(Y_i)\|_\infty \le 2\|f\|_\infty$. Scale both sides by a factor of $\sqrt{n}$ and we get

$$\mathbb{P}(\left|\sqrt{n}\mathbb{M}_n(f)\right| > t) \le 2\exp\left\{ -\frac{1}{2} \frac{t^2}{\|\langle M \rangle_n\|_\infty / n + 2\|f\|_\infty t/(3\sqrt{n})} \right\}, \tag{10}$$

where $t > 0$ and $\langle M \rangle_n = \sum_{i=1}^n \operatorname{Var}[f(Y_i)|\mathcal{G}_{i-1}]$. The result follows by applying the inequality (10) to Lemma 15 and expand the L_ψ-Orlicz norm. ■

The following lemma shows how the dependence of π_i on $\hat{f}_{i-1}$ can be canceled by the sampling probability so that it reduces to a constant term.

Lemma 17 *Under our problem settings, remember that the covariates $\boldsymbol{X}_i$ are i.i.d. Besides, $\mathcal{G}_i$ is defined as $\sigma\{\boldsymbol{H}_i\}, i \in \mathbb{N}$ and $\hat{f}_{i-1}$ is the estimated ITR based on $\boldsymbol{H}_{i-1}$. For any function $G : \mathcal{X} \mapsto \mathbb{R}$, we have*

$$\mathbb{E}\left[\frac{G(\boldsymbol{X}_i)}{\pi_i(A_i; \boldsymbol{H}_{i-1}, \boldsymbol{X}_i)}\right] = \mathbb{E}\left[\frac{G(\boldsymbol{X}_i)}{\pi_i(A_i; \boldsymbol{H}_{i-1}, \boldsymbol{X}_i)}\middle|\mathcal{G}_{i-1}\right] = 2\mathbb{E}[G(\boldsymbol{X}_i)], \tag{11}$$

$$\mathbb{E}\left[\frac{G(\boldsymbol{X}_i)}{\pi_i^2(A_i; \boldsymbol{H}_{i-1}, \boldsymbol{X}_i)}\right] = \mathbb{E}\left[\frac{G(\boldsymbol{X}_i)}{\pi_i^2(A_i; \boldsymbol{H}_{i-1}, \boldsymbol{X}_i)}\middle|\mathcal{G}_{i-1}\right] \le \left(\frac{1}{\epsilon_i} + \frac{1}{0.5}\right)\mathbb{E}[G(\boldsymbol{X}_i)]. \tag{12}$$

Proof For the first equation (11), notice that by tower property and the definition of I_i,

$$\begin{aligned}
&\mathbb{E}\left[\frac{G(\boldsymbol{X}_i)}{\pi_i(A_i; \boldsymbol{H}_{i-1}, \boldsymbol{X}_i)}\middle|\mathcal{G}_{i-1}\right] \\
=&\mathbb{E}\left\{ G(\boldsymbol{X}_i)\mathbb{E}\left[\frac{\mathbb{1}(I_i = 1)}{\pi_i(\hat{f}_{i-1}(\boldsymbol{X}_i); \boldsymbol{H}_{i-1}, \boldsymbol{X}_i)} + \frac{\mathbb{1}(I_i = -1)}{\pi_i(-\hat{f}_{i-1}(\boldsymbol{X}_i); \boldsymbol{H}_{i-1}, \boldsymbol{X}_i)}\middle|\boldsymbol{X}_i, \mathcal{G}_{i-1}\right]\middle|\mathcal{G}_{i-1}\right\}
\end{aligned}$$

We have that $\pi_i(\hat{f}_{i-1}(\boldsymbol{X}_i); \boldsymbol{H}_{i-1}, \boldsymbol{X}_i)$ is a fixed function of $\boldsymbol{H}_{i-1}, \boldsymbol{X}_i$ and that it equals $\mathbb{E}[\mathbb{1}(I_i = 1) | \boldsymbol{H}_{i-1}, \boldsymbol{X}_i]$. It is also true for the second term in the bracket. Therefore, the right-hand side equals $\mathbb{E}[2G(\boldsymbol{X}_i)|\mathcal{G}_{i-1}]$. The result follows from the assumption that $\boldsymbol{X}_i$ is independent of the history. The first equality in (11) can be proved by taking expectation of both sides of the equation.

Similarly, when G is divided by the square term of π_i in (12),

$$
\begin{aligned}
&\mathbb{E}\left[\frac{G(\boldsymbol{X}_i)}{\pi_i^2(A_i; \boldsymbol{H}_{i-1}, \boldsymbol{X}_i)}\middle|\mathcal{G}_{i-1}\right] \\
=&\mathbb{E}\left\{G(\boldsymbol{X}_i)\mathbb{E}\left[\frac{\mathbb{1}(I_i = 1)}{\pi_i^2(\hat{f}_{i-1}(\boldsymbol{X}_i); \boldsymbol{H}_{i-1}, \boldsymbol{X}_i)} + \frac{\mathbb{1}(I_i = -1)}{\pi_i^2(-\hat{f}_{i-1}(\boldsymbol{X}_i); \boldsymbol{H}_{i-1}, \boldsymbol{X}_i)}\middle|\boldsymbol{X}_i, \mathcal{G}_{i-1}\right]\middle|\mathcal{G}_{i-1}\right\} \\
=&\mathbb{E}\left\{G(\boldsymbol{X}_i)\left[\frac{1}{\pi_i(\hat{f}_{i-1}(\boldsymbol{X}_i); \boldsymbol{H}_{i-1}, \boldsymbol{X}_i)} + \frac{1}{\pi_i(-\hat{f}_{i-1}(\boldsymbol{X}_i); \boldsymbol{H}_{i-1}, \boldsymbol{X}_i)}\right]\middle|\mathcal{G}_{i-1}\right\}.
\end{aligned}
$$

Since one of $\pi_i(\hat{f}_{i-1}(\boldsymbol{X}_i); \boldsymbol{H}_{i-1}, \boldsymbol{X}_i)$ and $\pi_i(-\hat{f}_{i-1}(\boldsymbol{X}_i); \boldsymbol{H}_{i-1}, \boldsymbol{X}_i)$ must be lower bounded by 0.5 and the other one is lower bounded by ϵ_i, the inequality follows. Now for the first term in (12), its upper bound can be proved by taking expectation of both sides of the inequality. ■

Appendix B. Proof of Lemma 6

The proof essentially follows the proof of Van der Vaart and Wellner (1996, Theorem 2.5.6), the bracketing entropy Donsker theorem, with an extension to martingale sequences.

In this proof, we assume that there exists a constant τ^2 such that the second conditional moment $\mathbb{E}(R^2|\boldsymbol{X}, A) \leq \tau^2$. This also implies that the conditional variance $\mathrm{Var}(R|\boldsymbol{X}, A)$ is bounded by τ^2. However, we will show that τ^2 does not appear in the dominating term of the final bound.

Proof Define $L_{2,\infty}(\mathbb{P})$ norm as $\|f\|_{\mathbb{P},2,\infty} = \sup_{x>0}[x^2\mathbb{P}(|f(\boldsymbol{X})| > x)]^{1/2}$. Note that $L_{2,\infty}(\mathbb{P})$ norm is not actually a norm, but it can be shown that there is a norm equivalent to it up to a constant multiple. The assumption (4) implies that

$$
\int_0^\infty \sqrt{\log N_{[]}(\eta, \mathcal{F}, L_{2,\infty}(\mathbb{P}))}d\eta + \int_0^\infty \sqrt{\log N(\eta, \mathcal{F}, L_2(\mathbb{P}))}d\eta < \infty,
$$

because $\|f\|_{\mathbb{P},2} \geq \|f\|_{\mathbb{P},2,\infty}$ for any measurable function f, and we have $N_{[]}(\eta, \mathcal{F}, L_2(\mathbb{P})) \geq N(\eta, \mathcal{F}, L_2(\mathbb{P}))$ for any function class $\mathcal{F}$.

For each positive integer q, define a bracketing number $N_q^1 := N_{[]}(2^{-q}, \mathcal{F}, L_{2,\infty}(\mathbb{P}))$ and a covering number $N_q^2 := N(2^{-q}, \mathcal{F}, L_2(\mathbb{P}))$. Then there are two partitions $\{\mathcal{F}_{qj}\}_{j=1}^{N_q^1}$ and $\{\mathcal{F}_{qk}\}_{k=1}^{N_q^2}$ of $\mathcal{F}$ into disjoint sets such that $\sum_q 2^{-q}\sqrt{\log N_q^1} < \infty$ and $\sum_q 2^{-q}\sqrt{\log N_q^2} < \infty$. Take intersection of the two partitions that correspond to the bracketing number and covering number respectively. The total number of sets will be $N_q := N_q^1 N_q^2$ and this joint

partition $\{\mathcal{F}_{qj}\}_{j=1}^{N_q}$ satisfies the combined conditions:

$$\sum_q 2^{-q}\sqrt{\log N_q} < \infty, \tag{13}$$

$$\left\|\left(\sup_{f,g\in\mathcal{F}_{qj}} |f-g|\right)^*\right\|_{\mathbb{P},2,\infty} < 2^{-q}, \quad \forall j \in \{1,\ldots,N_q\}, \tag{14}$$

$$\sup_{f,g\in\mathcal{F}_{qj}} \|f-g\|_{\mathbb{P},2} < 2^{-q}, \quad \forall j \in \{1,\ldots,N_q\}. \tag{15}$$

Furthermore, the sequence of partitions can be chosen to be nested. To see this, consider a sequence of partitions $\{\bar{\mathcal{F}}_{qj}\}_{j=1}^{\bar{N}_q}$ that are possibly not nested. Take the partition at stage q to consist of all intersections of the form $\bigcap_{p=1}^{q} \bar{\mathcal{F}}_{p,i_p}$. Then this generates $N_q = \bar{N}_1 \ldots \bar{N}_q$ sets. Conditions (13) - (15) continue to hold since $(\log \prod_{p=1}^{q} \bar{N}_p)^{1/2} \le \sum_{p=1}^{q} (\log \bar{N}_p)^{1/2}$.

Now for each q, fix a function $f_{qj} \in \mathcal{F}_{qj}$ to be the representative of the set $\mathcal{F}_{qj}$ and let ξ be the function of choosing the representative. In addition, let Δ be the function of finding the "size" of the set that a function belongs to. Then we have

$$\xi_q f := \sum_j I(f \in \mathcal{F}_{qj}) f_{qj}, \quad \text{and} \quad \Delta_q f := \sum_j I(f\in\mathcal{F}_{qj}) \sup_{f_1,f_2\in\mathcal{F}_{qj}} |f_1 - f_2|^*.$$

For the weighted function h^f of f, define

$$\xi_q h^f := h^{\xi_q f}, \quad \text{and} \quad \Delta_q h^f := \sum_j I(f\in\mathcal{F}_{qj}) \sup_{f_1,f_2\in\mathcal{F}_{qj}} \left|h^{f_1} - h^{f_2}\right|^*.$$

Note that $\Delta_q h^f = \sum_j I(f \in \mathcal{F}_{qj}) \sup_{f_1,f_2\in\mathcal{F}_{qj}} \left|g^{f_1} - g^{f_2}\right|^*$ and $|\phi(Af_1) - \phi(Af_2)| \le |f_1 - f_2|$ since $\phi(Af)$ is Lipschitz 1 with respect to f. Hence we obtain that $|R_i|\,\Delta_q h^f(\boldsymbol{Z}_i) \le \Delta_q f(\boldsymbol{X}_i)/\pi_i(A_i;\hat{f}_{i-1})$. Note that $\xi_q h^f$ and $\Delta_q h^f$ form sets of only N_q functions when h^f ranges over $\mathcal{F}$. We will actually approximate each h^f with $\xi_q h^f$ and $\Delta_q h^f$. While $\mathcal{F}$ may be infinite, $\xi_q h^f$ and $\Delta_q h^f$ run over finite sets.

Let $\mathrm{Log}(x) := 1 + \log(x)$. For each fixed n and q_0, define truncation levels a_q and indicator functions A_q, B_q for $q \ge q_0$ as

$$\begin{aligned}
a_q &= 2^{-q}/\sqrt{\mathrm{Log}\, N_{q+1}}, \quad \forall q \ge q_0,\\
A_{q-1} f &= \mathbb{1}\left\{\Delta_{q_0} f \le \sqrt{n} a_{q_0}, \ldots, \Delta_{q-1} f \le \sqrt{n} a_{q-1}\right\}, \quad \forall q > q_0,\\
B_q f &= A_{q-1} f \mathbb{1}\left\{\Delta_q f > \sqrt{n} a_q\right\}, \quad \forall q > q_0,\\
B_{q_0} f &= \mathbb{1}\left\{\Delta_{q_0} f > \sqrt{n} a_{q_0}\right\}.
\end{aligned}$$

Since the partitions are nested, the functions A_q and B_q are constants in f on each set $\mathcal{F}_{qj}$ in level q. The key observation here is that

$$h^f - \xi_{q_0} h^f = (h^f - \xi_{q_0} h^f) B_{q_0} f + \sum_{q=q_0+1}^{\infty} (h^f - \xi_q h^f) B_q f + \sum_{q=q_0+1}^{\infty} (\xi_q h^f - \xi_{q-1} h^f) A_{q-1} f \tag{16}$$

pointwise in x. To see this, note that either $B_q f = 0$ for all q or there is a unique q_1 such that $B_q f = 1$. In the former case, the first two terms are all zero and the third term has

canceling components and converges to $f - \xi_{q_0} f$. In the latter case, the right-hand side of (16) is equivalent to $h^f - \xi_{q_1} h^f + \sum_{q=q_0+1}^{q_1} (\xi_q h^f - \xi_{q-1} h^f)$, and the result follows.

Write $\|\mathbb{M}_n(f)\|_{\mathcal{F}}$ as the supremum of $|\mathbb{M}_n(f)|$ as f ranges over $\mathcal{F}$. Then $\mathbb{E}^* \sup_{f\in\mathcal{F}} \mathbb{W}_n(f)$ can be bounded as

$$\begin{aligned}
\mathbb{E}^* \left\| \sqrt{n}\mathbb{W}_n(f) \right\|_{\mathcal{F}} \le & \mathbb{E}^* \left\| \sqrt{n}\mathbb{M}_n(h^f - \xi_{q_0} h^f) B_{q_0} f \right\|_{\mathcal{F}} && (17)\\
& + \mathbb{E}^* \left\| \sum_{q=q_0+1}^{\infty} \sqrt{n}\mathbb{M}_n(h^f - \xi_q h^f) B_q f \right\|_{\mathcal{F}} && (18)\\
& + \mathbb{E}^* \left\| \sum_{q=q_0+1}^{\infty} \sqrt{n}\mathbb{M}_n(\xi_q h^f - \xi_{q-1} h^f) A_{q-1} f \right\|_{\mathcal{F}} && (19)\\
& + \mathbb{E}^* \left\| \sqrt{n}\mathbb{M}_n \xi_{q_0} h^f \right\|_{\mathcal{F}}. && (20)
\end{aligned}$$

To bound the first term (17), note that for any function class $\mathcal{H}$ with some envelope function H, $|\mathbb{M}_n(h)| \le \frac{1}{n}\sum_{i=1}^n (\mathbb{E}_{i-1} H(\boldsymbol{Z}_i) + H(\boldsymbol{Z}_i))$ for all $h \in \mathcal{H}$. Then we have

$$\mathbb{E}^* \|\mathbb{M}_n(h)\|_{\mathcal{H}} \le \mathbb{E}^* \left\| \frac{1}{n}\sum_{i=1}^n \mathbb{E}_{i-1} H(\boldsymbol{Z}_i) + H(\boldsymbol{Z}_i) \right\|_{\mathcal{H}} = \frac{2}{n}\sum_{i=1}^n \mathbb{E}^* H(\boldsymbol{Z}_i).$$

An envelope function of $(h^f - \xi_{q_0} h^f) B_{q_0} f$ is

$$\frac{|R_i|}{\pi_i(A_i; \boldsymbol{H}_{i-1}, \boldsymbol{X}_i)} \left| h^f - \xi_{q_0} h^f \right| B_{q_0} f \le \frac{r}{\pi_i(A_i; \boldsymbol{H}_{i-1}, \boldsymbol{X}_i)} 2F \mathbb{1}\left\{2F > \sqrt{n} a_{q_0}\right\}$$

by the definitions of envelope function F and indicator function B_{q_0}. Therefore,

$$\begin{aligned}
\mathbb{E}^* \left\| \sqrt{n}\mathbb{M}_n(h^f - \xi_{q_0} h^f) B_{q_0} f \right\|_{\mathcal{F}} \le & \frac{2}{\sqrt{n}} \sum_{i=1}^n \mathbb{E}^* \frac{r}{\pi_i(A_i; \boldsymbol{H}_{i-1}, \boldsymbol{X}_i)} 2F(\boldsymbol{X}_i) \mathbb{1}\left\{2F(\boldsymbol{X}_i) > \sqrt{n} a_{q_0}\right\}\\
= & \frac{4r}{\sqrt{n}} \sum_{i=1}^n \mathbb{E}^* 2F(\boldsymbol{X}_i) \mathbb{1}\left\{2F(\boldsymbol{X}_i) > \sqrt{n} a_{q_0}\right\}\\
\le & \frac{4r}{a_{q_0}} \mathbb{E}^* \left[(2F)^2 \mathbb{1}\left\{2F > \sqrt{n} a_{q_0}\right\}\right]\\
\lesssim & \frac{r}{a_{q_0}} \|F\|_{\mathbb{P},2}^2 .
\end{aligned}$$

The equality comes from Lemma 17 and the third line is true since $\boldsymbol{X}_i$'s are i.i.d. Choose q_0 such that $2^{-q_0} = \delta \|F\|_{\mathbb{P},2}$ for some $\delta > 0$. Then

$$\mathbb{E}^* \left\| \sqrt{n}\mathbb{M}_n(h^f - \xi_{q_0} h^f) B_{q_0} f \right\|_{\mathcal{F}} \lesssim r 2^{-q_0} \sqrt{\operatorname{Log} N_{q_0}}. \tag{21}$$

For any function class $\mathcal{H}$ with some envelope function H, we can bound $|\mathbb{M}_n h|$ by $\frac{1}{n}\sum_{i=1}^n (\mathbb{E}_{i-1} H + H) = -\mathbb{M}_n H + \frac{2}{n}\sum_{i=1}^n \mathbb{E}_{i-1} H$ for any $h \in \mathcal{H}$. Since $\left|(h^f - \xi_q h^f) B_q f\right| \le$

$|R_i|\,\Delta_q f B_q f/\pi_i(A_i;\hat{f}_{i-1})$, the second term (18) can be bounded by

$$\begin{aligned}
&\mathbb{E}^*\left\|\sum_{q=q_0+1}^{\infty}\sqrt{n}\mathbb{M}_n(h^f-\xi_q h^f)B_q f\right\|_{\mathcal{F}}\\
&=\sum_{q=q_0+1}^{\infty}\mathbb{E}^*\left\|\sqrt{n}\mathbb{M}_n\frac{|R_i|}{\pi_i(A_i;\boldsymbol{H}_{i-1},\boldsymbol{X}_i)}\Delta_q f B_q f\right\|_{\mathcal{F}} \qquad (22)\\
&\quad+\sum_{q=q_0+1}^{\infty}2\sqrt{n}\mathbb{E}^*\left\|\frac{1}{n}\sum_{i=1}^{n}\mathbb{E}_{i-1}\frac{|R_i|}{\pi_i(A_i;\boldsymbol{H}_{i-1},\boldsymbol{X}_i)}\Delta_q f B_q f\right\|_{\mathcal{F}}.
\end{aligned}$$

By Corollary 16, for each q in the first term in (22), the expectation can be split into two parts:

$$\begin{aligned}
&\mathbb{E}^*\left\|\sqrt{n}\mathbb{M}_n\frac{|R_i|}{\pi_i(A_i;\boldsymbol{H}_{i-1},\boldsymbol{X}_i)}\Delta_q f B_q f\right\|_{\mathcal{F}}\\
&\lesssim\frac{1}{\sqrt{n}}\max_{f\in\mathcal{F}}\left\|\frac{|R_i|}{\pi_i(A_i;\boldsymbol{H}_{i-1},\boldsymbol{X}_i)}\Delta_q f B_q f\right\|_{\infty}\operatorname{Log}N_q\\
&\quad+\frac{1}{\sqrt{n}}\max_{f\in\mathcal{F}}\sqrt{\left\|\sum_{i=1}^{n}\operatorname{Var}\left[\frac{|R_i|}{\pi_i(A_i;\boldsymbol{H}_{i-1},\boldsymbol{X}_i)}\Delta_q f B_q f\middle|\mathcal{G}_{i-1}\right]\right\|_{\infty}}\sqrt{\operatorname{Log}N_q}.
\end{aligned}$$

Since $\Delta_q f B_q f\le\Delta_{q-1}fA_{q-1}f\le\sqrt{n}a_{q-1}$, the L_∞ term in the first part can be bounded by

$$\left\|\frac{|R_i|}{\pi_i(A_i;\boldsymbol{H}_{i-1},\boldsymbol{X}_i)}\Delta_q f B_q f\right\|_{\infty}\le\frac{r}{\epsilon_n}\sqrt{n}a_{q-1} \qquad (23)$$

for any $f\in\mathcal{F}$. For the second part, by the assumption of the second conditional moment,

$$\begin{aligned}
&\left\|\sum_{i=1}^{n}\operatorname{Var}\left[\frac{|R_i|}{\pi_i(A_i;\boldsymbol{H}_{i-1},\boldsymbol{X}_i)}\Delta_q f B_q f\middle|\mathcal{G}_{i-1}\right]\right\|_{\infty}\\
&\le\sum_{i=1}^{n}\left\|\mathbb{E}\left[\frac{\tau^2}{\pi_i^2(A_i;\boldsymbol{H}_{i-1},\boldsymbol{X}_i)}\Delta_q^2 f B_q^2 f\middle|\mathcal{G}_{i-1}\right]\right\|_{\infty} \qquad (24)\\
&\le\sum_{i=1}^{n}\left\|\left(\frac{1}{\epsilon_i}+\frac{1}{0.5}\right)\mathbb{E}\left(\tau^2\Delta_q^2 f B_q^2 f\right)\right\|_{\infty}.
\end{aligned}$$

The last inequality comes from Lemma 17. For any non-negative random variable X, we have the inequality $\|X\|_{2,\infty}^2\le\sup_{t>0}t\mathbb{E}\left[X\mathbb{1}(X>t)\right]\le 2\|X\|_{2,\infty}^2$. Then

$$\sqrt{n}a_q\mathbb{E}\left(\Delta_q f B_q f\right)\le\sqrt{n}a_q\mathbb{E}\left(\Delta_q f\mathbb{1}(\Delta_q f>\sqrt{n}a_q)\right)\le 2\|\Delta_q f\|_{\mathbb{P},2,\infty}^2\le 2\cdot 2^{-2q}. \qquad (25)$$

Since $\Delta_q f B_q f$ is bounded by $\sqrt{n}a_{q-1}$ for $q>q_0$, it follows that

$$\mathbb{E}\left(\Delta_q^2 f B_q^2 f\right)\le\sqrt{n}a_{q-1}\mathbb{E}\left(\Delta_q f B_q f\right)\le 2\frac{a_{q-1}}{a_q}2^{-2q}.$$

Using Lemma 17 again and the inequality (25), the second term in (22) can be bounded as

$$\begin{aligned}&\mathbb{E}^*\left\|\frac{1}{n}\sum_{i=1}^{n}\mathbb{E}_{i-1}\frac{|R_i|}{\pi_i(A_i;\boldsymbol{H}_{i-1},\boldsymbol{X}_i)}\Delta_q f B_q f\right\|_{\mathcal{F}}\\ \leq&\frac{1}{n}\sum_{i=1}^{n}\mathbb{E}^*\left\|2r\mathbb{E}\left(\Delta_q f B_q f\right)\right\|_{\mathcal{F}}\leq 4r\frac{1}{\sqrt{n}a_{q-1}}2^{-2q}.\end{aligned}\tag{26}$$

Now apply the above bounds (23), (24), (26) on (18) to find

$$\begin{aligned}&\mathbb{E}^*\left\|\sum_{q=q_0+1}^{\infty}\sqrt{n}\mathbb{M}_n(h^f-\xi_q h^f)B_q f\right\|_{\mathcal{F}}\\ \lesssim&\sum_{q=q_0+1}^{\infty}\Bigg[\frac{1}{\sqrt{n}}\frac{r}{\epsilon_n}\sqrt{n}a_{q-1}\operatorname{Log}N_q+\frac{1}{\sqrt{n}}\sqrt{2\tau^2\sum_{i=1}^{n}\left(\frac{1}{\epsilon_i}+\frac{1}{0.5}\right)\frac{a_{q-1}}{a_q}2^{-2q}}\sqrt{\operatorname{Log}N_q}\\ &\qquad+\sqrt{n}4r\frac{1}{\sqrt{n}a_{q-1}}2^{-2q}\Bigg]\\ \lesssim&\sum_{q=q_0+1}^{\infty}\left[\frac{r}{\epsilon_n}+\tau\sqrt{\frac{1}{n}\sum_{i=1}^{n}\frac{1}{\epsilon_i}}+r\right]2^{-q}\sqrt{\operatorname{Log}N_q}.\end{aligned}\tag{27}$$

The last inequality comes from the fact that $a_{q-1}/a_q\leq(a_{q-1}/a_q)^2$ for decreasing a_q.

To handle the third term (19), first note that it is bounded by

$$\sum_{q=q_0+1}^{\infty}\mathbb{E}^*\left\|\sqrt{n}\mathbb{M}_n\frac{|R_i|}{\pi_i(A_i;\boldsymbol{H}_{i-1},\boldsymbol{X}_i)}\Delta_{q-1}fA_{q-1}f\right\|_{\mathcal{F}},$$

since the partition is nested. Then we can use Corollary 16 as in the bound of the second term (18). The maximum of L_∞ norm over $\mathcal{F}$ in the first part is upper bounded by $r\sqrt{n}a_{q-1}/\epsilon_n$. For the second part, use Lemma 17 and assumption (15) to find

$$\begin{aligned}&\left\|\sum_{i=1}^{n}\operatorname{Var}\left[\frac{|R_i|}{\pi_i(A_i;\boldsymbol{H}_{i-1},\boldsymbol{X}_i)}\Delta_{q-1}fA_{q-1}f\middle|\mathcal{G}_{i-1}\right]\right\|_{\infty}\\ \leq&\left\|\sum_{i=1}^{n}\mathbb{E}\left[\frac{\tau^2}{\pi_i^2(A_i;\boldsymbol{H}_{i-1},\boldsymbol{X}_i)}\Delta_{q-1}^2 f\middle|\mathcal{G}_{i-1}\right]\right\|_{\infty}\\ \leq&\tau^2\sum_{i=1}^{n}\left(\frac{1}{\epsilon_i}+\frac{1}{0.5}\right)\left\|\mathbb{E}\Delta_{q-1}^2 f\right\|_{\infty}\\ \leq&\tau^2\sum_{i=1}^{n}\left(\frac{1}{\epsilon_i}+\frac{1}{0.5}\right)(2^{-q+1})^2.\end{aligned}$$

Combining the two parts together, we have

$$\begin{aligned}
&\mathbb{E}^*\left\|\sum_{q=q_0+1}^{\infty}\sqrt{n}\mathbb{M}_n(\xi_q h^f-\xi_{q-1}h^f)A_{q-1}f\right\|_{\mathcal{F}}\\
&\lesssim\sum_{q=q_0+1}^{\infty}\left[\frac{1}{\sqrt{n}}\frac{r}{\epsilon_n}\sqrt{n}a_{q-1}\operatorname{Log}N_q+\frac{1}{\sqrt{n}}\sqrt{2\tau^2\sum_{i=1}^{n}\left(\frac{1}{\epsilon_i}+\frac{1}{0.5}\right)2^{-2q+2}\sqrt{\operatorname{Log}N_q}}\right] \qquad (28)\\
&\lesssim\sum_{q=q_0+1}^{\infty}\left[\frac{r}{\epsilon_n}+\tau\sqrt{\frac{1}{n}\sum_{i=1}^{n}\frac{1}{\epsilon_i}}\right]2^{-q}\sqrt{\operatorname{Log}N_q}.
\end{aligned}$$

For the last term (20), consider two cases on whether the envelope function F is bounded by $\sqrt{n}a_{q_0}$ or not. Apply Corollary 16 in the first case. With the supremum part bounded by

$$\left\|\xi_{q_0}h^f\mathbb{1}\left(F\le\sqrt{n}a_{q_0}\right)\right\|_\infty\le\frac{r}{\epsilon_n}\left\|2F\mathbb{1}\left(F\le\sqrt{n}a_{q_0}\right)\right\|_\infty\le\frac{2r}{\epsilon_n}\sqrt{n}a_{q_0}$$

and the conditional variance part bounded by

$$\left\|\sum_{i=1}^{n}\operatorname{Var}\left(\xi_{q_0}h\mathbb{1}\left(F\le\sqrt{n}a_{q_0}\right)|\mathcal{G}_{i-1}\right)\right\|_\infty\le\tau^2\sum_{i=1}^{n}\left(\frac{1}{1-\epsilon_i}+\frac{1}{\epsilon_i}\right)\|F\|_{\mathbb{P},2}^2,$$

we have

$$\begin{aligned}
&\mathbb{E}^*\left\|\sqrt{n}\mathbb{M}_n\xi_{q_0}h^f\mathbb{1}\left(F\le\sqrt{n}a_{q_0}\right)\right\|_{\mathcal{F}}\\
\lesssim&\frac{1}{\sqrt{n}}\frac{r}{\epsilon_n}\sqrt{n}a_{q_0}\operatorname{Log}N_{q_0}+\frac{1}{\sqrt{n}}\sqrt{\tau^2\sum_{i=1}^{n}\left(\frac{1}{\epsilon_i}+\frac{1}{0.5}\right)\|F\|_{\mathbb{P},2}^2\sqrt{\operatorname{Log}N_{q_0}}}\\
\lesssim&\frac{r}{\epsilon_n}2^{-q_0}\sqrt{\operatorname{Log}N_{q_0}}+\tau\sqrt{\frac{1}{n}\sum_{i=1}^{n}\frac{1}{\epsilon_i}}\|F\|_{\mathbb{P},2}\sqrt{\operatorname{Log}N_{q_0}}.
\end{aligned}$$

In the second case, since $\xi_{q_0}h^f\mathbb{1}\left(F>\sqrt{n}a_{q_0}\right)$ is bounded by $\frac{2r}{\epsilon_n}F\mathbb{1}\left(F>\sqrt{n}a_{q_0}\right)$,

$$\mathbb{E}^*\left\|\sqrt{n}\mathbb{M}_n\xi_{q_0}h^f\mathbb{1}\left(F>\sqrt{n}a_{q_0}\right)\right\|_{\mathcal{F}}\lesssim\frac{r}{a_{q_0}}\|F\|_{\mathbb{P},2}^2$$

by the same argument in the bounds for (21). Therefore, by applying the triangle inequality and choosing q_0 so that $2^{-q_0}=\delta\|F\|_{\mathbb{P},2}$ for some constant $\delta>0$,

$$\begin{aligned}
&\mathbb{E}^*\left\|\sqrt{n}\mathbb{M}_n\xi_{q_0}h^f\right\|_{\mathcal{F}}\\
\lesssim&\frac{r}{\epsilon_n}2^{-q_0}\sqrt{\operatorname{Log}N_{q_0}}+\tau\sqrt{\frac{1}{n}\sum_{i=1}^{n}\frac{1}{\epsilon_i}}\|F\|_{\mathbb{P},2}\sqrt{\operatorname{Log}N_{q_0}}+\frac{r}{a_{q_0}}\|F\|_{\mathbb{P},2}^2 \qquad (29)\\
\lesssim&\left[\frac{r}{\epsilon_n}+\tau\sqrt{\frac{1}{n}\sum_{i=1}^{n}\frac{1}{\epsilon_i}}+r\right]2^{-q_0}\sqrt{\operatorname{Log}N_{q_0}},
\end{aligned}$$

where $N_{q_0} = N_{[]}(\delta \|F\|_{\mathbb{P},2}, \mathcal{F}, L_2(\mathbb{P}))$.

Finally, combine the four upper bounds (21), (27), (28) and (29) together to get

$$\begin{aligned}\mathbb{E}^* \left\|\sqrt{n}\mathbb{W}_n(f)\right\|_{\mathcal{F}} \lesssim& \sum_{q=q_0}^{\infty} \left[\frac{r}{\epsilon_n} + \tau\sqrt{\frac{1}{n}\sum_{i=1}^{n}\frac{1}{\epsilon_i}} + r\right] 2^{-q}\sqrt{\operatorname{Log} N_q} \\ \lesssim& \frac{r}{\epsilon_n} J_{[]}(\|F\|_{\mathbb{P},2}, \mathcal{F}, L_2(\mathbb{P})).\end{aligned}$$

■

Appendix C. Proof of Theorem 1

Using Lemma 13 and Lemma 6, we can give our proof of Theorem 1. Apart from applying the bound on the expectation of supremum to the concentration inequality of martingale process, we also combine the pilot trial with the main trial which follows the adaptive design.

Proof First note that $\mathcal{V}(f^*) - \mathcal{V}(f) = \mathbb{E}^{\operatorname{sign}\{f^*\}}(R) - \mathbb{E}^{\operatorname{sign}\{f\}}(R)$, which is the opposite of excess 0-1 risk. For the initial n_0 i.i.d. observations, we know that the excess 0-1 risk is bounded by excess ϕ-risk, that is,

$$\mathbb{E}h^f(\boldsymbol{Z}_i^{(0)}) \geq \mathbb{E}^{\operatorname{sign}\{f^*\}}(R_i) - \mathbb{E}^{\operatorname{sign}\{f\}}(R_i)$$

for any $i = 1, \ldots, n_0$ and any measurable f by Theorem 3.2 in Zhao et al. (2012). For sequentially generated data $\{\boldsymbol{Z}_i\}_{i=1}^n$, note that conditioning on $\mathcal{G}_{i-1}$,

$$\begin{aligned}\mathbb{E}_{i-1}h^f(\boldsymbol{Z}_i) =& \mathbb{E}\left(\frac{R_i\phi(A_i f)}{\pi_i(A_i;\boldsymbol{H}_{i-1},\boldsymbol{X}_i)}\middle|\mathcal{G}_{i-1}\right) - \mathbb{E}\left(\frac{R_i\phi(A_i f^*)}{\pi_i(A_i;\boldsymbol{H}_{i-1},\boldsymbol{X}_i)}\middle|\mathcal{G}_{i-1}\right) \\ \geq& \mathbb{E}\left(\frac{R_i\mathbb{1}\{A_i \neq \operatorname{sign}\{f\}\}}{\pi_i(A_i;\boldsymbol{H}_{i-1},\boldsymbol{X}_i)}\middle|\mathcal{G}_{i-1}\right) - \mathbb{E}\left(\frac{R_i\mathbb{1}\left\{A_i \neq \operatorname{sign}\{f^*\}\right\}}{\pi_i(A_i;\boldsymbol{H}_{i-1},\boldsymbol{X}_i)}\middle|\mathcal{G}_{i-1}\right) \\ =& \mathbb{E}^{\operatorname{sign}\{f^*\}}(R_i) - \mathbb{E}^{\operatorname{sign}\{f\}}(R_i)\end{aligned}$$

for any $i = 1, \ldots, n$ and any measurable f. The inequality can be proved similarly as in the i.i.d. case of Theorem 3.2 in Zhao et al. (2012), but with a condition on $\mathcal{G}_{i-1}$. Therefore, the value function difference $\mathcal{V}(f^*) - \mathcal{V}(\hat{f}_n)$ is upper bounded by

$$\frac{1}{n_0+n}\left[\sum_{i=1}^{n_0}\mathbb{E}h^{\hat{f}_n}(\boldsymbol{Z}_i^{(0)}) + \sum_{i=1}^{n}\mathbb{E}_{i-1}h^{\hat{f}_n}(\boldsymbol{Z}_i)\right].$$

In SRAT, $\hat{f}_n$ should be minimizing $\sum_{i=1}^{n_0} g^f(\boldsymbol{Z}_i^{(0)}) + \sum_{i=1}^{n} g^f(\boldsymbol{Z}_i)$, so we have

$$\sum_{i=1}^{n_0} h^{\hat{f}_n}(\boldsymbol{Z}_i^{(0)}) + \sum_{i=1}^{n} h^{\hat{f}_n}(\boldsymbol{Z}_i) \leq 0.$$

It follows that

$$\begin{aligned} &\mathcal{V}(f^*) - \mathcal{V}(\hat{f}_n) \\ \leq& \frac{1}{n_0+n}\left[\sum_{i=1}^{n_0}\mathbb{E}h^{\hat{f}_n}(\boldsymbol{Z}_i^{(0)}) + \sum_{i=1}^{n}\mathbb{E}_{i-1}h^{\hat{f}_n}(\boldsymbol{Z}_i) - \sum_{i=1}^{n_0}h^{\hat{f}_n}(\boldsymbol{Z}_i^{(0)}) - \sum_{i=1}^{n}h^{\hat{f}_n}(\boldsymbol{Z}_i)\right] \\ \leq& \sup_{f\in\mathcal{F}}\frac{1}{n_0+n}\left[\sum_{i=1}^{n_0}\left[\mathbb{E}h^f(\boldsymbol{Z}_i^{(0)}) - h^f(\boldsymbol{Z}_i^{(0)})\right] + \sum_{i=1}^{n}\left[\mathbb{E}_{i-1}h^f(\boldsymbol{Z}_i) - h^f(\boldsymbol{Z}_i)\right]\right]. \end{aligned} \tag{30}$$

Now it suffices to bound the right-hand side of (30).

We will use Lemma 13 to bound the martingale part. First we test the conditions of the lemma. Since $\mathcal{R}_n(\mathcal{F})$ and $\sup_{\mathbb{P}}\mathbb{E}\sup_{f\in\mathcal{F}}\mathbb{M}_n(f)$ are on the same scale and the latter one $\sup_{\mathbb{P}}\mathbb{E}\sup_{f\in\mathcal{F}}\mathbb{M}_n(f)$ is in the order of $1/\sqrt{n}$, the first assumption in Lemma 13 is satisfied. The second one can be satisfied when taking a large class $\mathcal{F}$, for example, a linear class with parameters bounded loosely.

Let $\mathcal{H}(\mathcal{F})$ be the class of functions constructed by h^f as f ranges over $\mathcal{F}$. According to (8),

$$\mathcal{R}_n(\mathcal{H}(\mathcal{F})) \leq 2\sup_{\mathbb{P}}\mathbb{E}\sup_{f\in\mathcal{F}}\mathbb{W}_n(f) + \frac{D}{2\sqrt{n}},$$

where $D = \inf_{z\in\mathcal{Z}}\sup_{h^f,h^{f'}\in\mathcal{F}}[h^f(z) - h^{f'}(z)] \geq 0$. Since R and h^f can take value zero, $D=0$ here. Therefore, $\mathcal{R}_n(\mathcal{H}(\mathcal{F}))$ is bounded by $rJ_{[]}(\|F\|_{\mathbb{P},2}, \mathcal{F}, L_2(\mathbb{P}))/(\sqrt{n}\epsilon_n)$ up to a constant by Lemma 6. Since $\mathbb{E}_{i-1}h^f(\boldsymbol{Z}_i) - h^f(\boldsymbol{Z}_i)$ is upper bounded by $2rb/\epsilon_n$ for all i and all $f\in\mathcal{F}$, scale (9) and we get

$$\mathbb{P}\left(\sup_{f\in\mathcal{F}}\left|\frac{1}{n}\sum_{i=1}^{n}\left[\mathbb{E}_{i-1}h^f(\boldsymbol{Z}_i) - h^f(\boldsymbol{Z}_i)\right]\right| > t\right) \leq 8L\exp\left\{-\frac{n\epsilon_n^4}{\log^3 n}\frac{t^2}{Cr^4b^2J^2}\right\}$$

for some constant C and any $t>0$. In other words,

$$\mathbb{P}\left(\sup_{f\in\mathcal{F}}\left|\sum_{i=1}^{n}\left[\mathbb{E}_{i-1}h^f(\boldsymbol{Z}_i) - h^f(\boldsymbol{Z}_i)\right]\right| > C\frac{r^2bJ}{\epsilon_n^2}\sqrt{n\log^3 n\delta}\right) \leq e^{-\delta} \tag{31}$$

for some constant C and any $\delta>0$.

To derive a bound for the initial randomized treatments of size n_0, we will take use of a variant of Talagrand's inequality (Talagrand, 1994) in Lemma 12, which is a common approach in i.i.d. classification problems. In our setting, $\mathbb{E}_{i-1}h^f(\boldsymbol{Z}_i) - h^f(\boldsymbol{Z}_i)$ has an expectation zero for all i and all $f\in\mathcal{F}$ and $\left\|\mathbb{E}_{i-1}h^f(\boldsymbol{Z}_i) - h^f(\boldsymbol{Z}_i)\right\|_\infty \leq 2rb/(1/2)$. Note that $\pi_i(A_i) = 1/2$ for all $i\in\{1,\ldots,n_0\}$ in pilot data. By assumption, $\frac{1}{n}\sum_{i=1}^{n}\sup_{f\in\mathcal{F}}\mathrm{Var}[h^f(\boldsymbol{Z}_i)]$ is bounded by $4b^2r^2$. The key step here is to bound

$$\mu^* = \mathbb{E}\left\{\sup_{f\in\mathcal{F}}\sum_{i=1}^{n_0}[\mathbb{E}h^f(\boldsymbol{Z}_i^{(0)}) - h^f(\boldsymbol{Z}_i^{(0)})]\right\}$$

in Lemma 12. By Theorem 2.14.2 in Van der Vaart and Wellner (1996), the expectation of supremum of an empirical process is bounded by the bracketing integral. Following

a similar proof of Lemma 6, with only Freedman's inequality (Freedman, 1975) replaced by Bernstein's inequality, we know $\mu^* \leq rJ\sqrt{n_0}$, since J is the supremum of bracketing integrals over all possible measures. Therefore,

$$\mathbb{P}\left(\sup_{f\in\mathcal{F}}\sum_{i=1}^{n_0}\left[\mathbb{E}h^f(\boldsymbol{Z}_i^{(0)}) - h^f(\boldsymbol{Z}_i^{(0)})\right] \geq 3rJ\sqrt{n_0} + \sqrt{4\delta br^2 n_0} + 4rb\delta\right) \leq e^{-\delta}. \tag{32}$$

Now by the triangle inequality and the fact that $\mathbb{P}(|X+Y| \geq a+b) \leq \mathbb{P}(|X| \geq a) + \mathbb{P}(|Y| \geq b)$,

$$\mathbb{P}\left(\sup_{f\in\mathcal{F}}\frac{1}{n_0+n}\left[\sum_{i=1}^{n_0}\left[\mathbb{E}h^f(\boldsymbol{Z}_i^{(0)}) - h^f(\boldsymbol{Z}_i^{(0)})\right] + \sum_{i=1}^{n}\left[\mathbb{E}_{i-1}h^f(\boldsymbol{Z}_i) - h^f(\boldsymbol{Z}_i)\right]\right]\right.$$
$$\left.\geq \frac{C}{n_0+n}\left[(rJ + r\sqrt{\delta b})\sqrt{n_0} + rb\delta + \frac{r^2 bJ}{\epsilon_n^2}\sqrt{n\log^3 n\delta}\right]\right) \leq e^{-\delta} \tag{33}$$

for some constant C and any $\delta > 0$. The result on test data follows by combining inequalities (30) and (33). ■

Appendix D. Proof of Theorem 7

Proof First note that

$$\left|\frac{1}{n}\sum_{i=1}^{n}\left[\mathcal{V}(\hat{f}_{i-1}) - R_i\right]\right| \leq \left|\frac{1}{n}\sum_{i=1}^{n}[R_i - \mathbb{E}_{i-1}R_i]\right| + \frac{1}{n}\sum_{i=1}^{n}\left|\mathbb{E}_{i-1}R_i - \mathcal{V}(\hat{f}_{i-1})\right|. \tag{34}$$

Given $\mathcal{G}_{i-1}$ and I_i, $\mathbb{E}(R_i|\mathcal{G}_{i-1}, I_i)$ is actually $\mathcal{V}(\hat{f}_{i-1}I_i)$. Then $\mathbb{E}_{i-1}R_i$ can be written as

$$\mathbb{E}\left[\mathbb{E}(R_i|\mathcal{G}_{i-1}, I_i)|\mathcal{G}_{i-1}\right] = \mathbb{E}[p_i(\boldsymbol{H}_{i-1}, \boldsymbol{X}_i)|\mathcal{G}_{i-1}]\mathcal{V}(\hat{f}_{i-1}) + \mathbb{E}[1 - p_i(\boldsymbol{H}_{i-1}, \boldsymbol{X}_i)|\mathcal{G}_{i-1}]\mathcal{V}(-\hat{f}_{i-1}),$$

where $p_i(\boldsymbol{H}_{i-1}, \boldsymbol{X}_i)$ is the probability of $I_i = 1$. So the second term of the right-hand side of (34) is upper bounded by

$$\frac{1}{n}\sum_{i=1}^{n}\mathbb{E}[1 - p_i(\boldsymbol{H}_{i-1}, \boldsymbol{X}_i)|\mathcal{G}_{i-1}]|\mathcal{V}(\hat{f}_{i-1}) - \mathcal{V}(-\hat{f}_{i-1})| \leq \frac{2r}{n}\sum_{i=1}^{n}\epsilon_i'.$$

For the first term, note that $\{R_i - \mathbb{E}_{i-1}R_i\}_{i=1}^n$ is a martingale difference sequence. We will use the Freedman's inequality in Lemma 14. The two parameters can be bounded as $\|R_i - \mathbb{E}_{i-1}R_i\|_\infty \leq 2r$ and $\left\|\sum_{i=1}^n \mathbb{E}_{i-1}(R_i - \mathbb{E}_{i-1})^2/n\right\|_\infty \leq nr^2$. Therefore,

$$\mathbb{P}\left(\left|\frac{1}{n}\sum_{i=1}^{n}[R_i - \mathbb{E}_{i-1}R_i]\right| \geq t\right) \leq 2\exp\left\{-\frac{1}{2}\frac{nt^2}{r^2 + 2rt/3}\right\}. \tag{35}$$

Let the right-hand side be $e^{-\delta}$ and the result follows.

■

Appendix E. Proof of Corollary 10

Proof First note that

$$\begin{aligned}&\left|\mathcal{V}(f^*) - \bar{R}_n\right| \\ \le& \left|\frac{1}{n}\sum_{i=1}^{n}\left[R_i - \mathbb{E}_{i-1}R_i\right]\right| + \frac{1}{n}\sum_{i=1}^{n}\left|\mathbb{E}_{i-1}R_i - \mathcal{V}(\hat{f}_{i-1})\right| + \frac{1}{n}\sum_{i=1}^{n}\left|\mathcal{V}(\hat{f}_{i-1}) - \mathcal{V}(f^*)\right|,\end{aligned}$$

where the first two terms are the same as in the decomposition (34). Now let the right-hand side of (35) be $e^{-\delta}/3$ and the right-hand side of (31) and (32) be $e^{-\delta}/3n$ by inverting the two bounds in the proof of Theorem 1. Note that in the third term we are comparing $\mathcal{V}(\hat{f}_i)$ with $\mathcal{V}(f^*)$ for $i = 0, \ldots, n-1$. When $i = 0$, the term in (31) does not actually exist. Hence letting $i \log^3 i = 0$ will work. ■

Appendix F. Proof of Theorem 11

Proof For the test regret bound (5), note that the last term

$$\frac{1}{n_0 + n}\frac{r^2 bJ}{\epsilon_n^2}\sqrt{\delta n \log^3 n}$$

is in the order of $O(n^{-1/2}(\log n)^{3/2}\epsilon_n^{-2})$ and the sum of the first two terms

$$\frac{1}{n_0 + n}\left[(J + \sqrt{\delta b})r\sqrt{n_0} + rb\delta\right]$$

is in the order of $O_p(n^{-1/2})$. So the last term dominates. In addition, we also want ϵ to be non-increasing and the last term to converge to 0. Therefore, θ should be no greater than 1 and larger than 0. Similarly, for the training regret bound (7), the second term that contains ϵ_n dominates. The result follows by substituting ϵ_n into the convergence rate and letting the two rates be equal. Note that

$$\frac{1}{n}\sum_{i=1}^{n}\epsilon_i = O(\left(\frac{1}{n}\int_0^n x^{-(1-\theta)/4}\mathrm{d}x\right) = O(n^{-(1-\theta)/4}).$$

■

Appendix G. Additional Simulation Results

Figure 7 of scenario 2 demonstrates similar results as Figure 2. However, since the dimension of predictors increases, the regret and false decision ratio of the test set are larger than that of scenario 1, especially for small sample sizes. LinUCB is not largely affected by the dimension compared to other methods. Therefore, SRATs with $\epsilon_0 = 0.1, \theta = 1$ exceed LinUCB on the test set only when n is larger than 600. RCT is better than AL-GP on the test set in this scenario, possibly because the nonparametric method is not efficient in a linear setting with a high dimension.
